# Novel Etoposide Analogue Modulates Expression of Angiogenesis Associated microRNAs and Regulates Cell Proliferation by Targeting STAT3 in Breast Cancer

**DOI:** 10.1371/journal.pone.0142006

**Published:** 2015-11-09

**Authors:** Chatla Srinivas, M. Janaki Ramaiah, A. Lavanya, Suresh Yerramsetty, P. B Kavi Kishor, Shaik Anver Basha, Ahmed Kamal, Utpal Bhadra, Manika-Pal Bhadra

**Affiliations:** 1 Centre for Chemical Biology, CSIR-Indian Institute of Chemical Technology, Hyderabad, India; 2 School of Chemical and Biotechnology, SASTRA University, Thanjavur, India; 3 Department of Genetics, Osmania University, Hyderabad, India; 4 Medicinal Chemistry and Pharmacology, CSIR-Indian Institute of Chemical Technology, Hyderabad, India; 5 Functional Genomics and Gene Silencing Group, CSIR-Centre for Cellular and Molecular Biology, Hyderabad, India; University of South Alabama, UNITED STATES

## Abstract

Tumor microenvironment play role in angiogenesis and carcinogenesis. Etoposide, a known topoisomerase II inhibitor induces DNA damage resulting in cell cycle arrest. We developed a novel Etoposide analogue, Quinazolino-4β-amidopodophyllotoxin (C-10) that show better efficacy in regulating cell proliferation and angiogenesis. We evaluated its role on expression of microRNAs-15, 16, 17 and 221 and its targets Bcl-2, STAT3 and VEGF that dictate cell proliferation and angiogenesis. Docking studies clearly demonstrated the binding of Etoposide and C-10 to STAT3. We conclude that combination of Etoposide or C-10 with miR-15, 16, 17 and 221 as a new approach to induce apoptosis and control angiogenesis in breast cancer.

## Introduction

Breast cancer is recognized as one of the most common type of cancers in women and its development is associated with risk factors such as alcohol consumption, diet and oral contraception [1]. Majority of breast cancers are estrogen receptor positive (ER+) and metastasis is the major reason for breast cancer related deaths [2]. Metastasis takes place due to genetic and epigenetic alterations. Cancer cells penetrate blood and lymph through intravascular system and proliferate in distant tissues whereby new vessels are formed by a process of angiogenesis. Therefore, neovascularisation is critical for tumor growth and metastasis which is triggered by signals from tumor cells [3]. The transition between latent to invasive (metastatic) phase of cancer is linked to an angiogenic switch. Onset of angiogenesis involves a balance between proangiogenic and antiangiogenic regulators of the tumor cells. Endothelial cell proliferation, migration and capillary tube formation are important events during angiogenesis. The expression of vascular endothelial growth factor (VEGF) by invasive tumors has been shown to correlate with vascularity and cell proliferation [4]. VEGF dependent signalling is found in both physiological and pathological vascular development and has been validated as a priority target for the development of anti and proangiogenic agents. Thus VEGF represents a critical inducer of tumour angiogenesis and targeting VEGF is the first choice of antiangiogenic therapies [5, 6].

The transcription factors STAT1 and STAT3 appear to play antagonistic roles in tumorigenesis. STAT3 promotes cell survival, proliferation, motility, immune tolerance and is considered as an oncogene, while STAT1 enhances inflammation, innate and adaptive immunity and triggers antiproliferative and proapoptotic responses in tumor cells [7]. Overexpression of STAT1 inhibits VEGF expression while STAT3 promotes VEGF expression [8–10]. Recent studies have suggested that antiapoptotic genes Bcl-2, Bcl-xL, Mcl-1 and angiogenic gene VEGF are regulated by STAT3 in various cancers [11, 12].

Small non-coding RNAs known as microRNAs (miRNAs) bind to 3’UTR of the target mRNAs and specifically inhibit translation. MicroRNAs have emerged as key players in cancer pathway by playing important roles in growth, metastasis, development and drug resistance [13, 14]. Specific microRNAs have been identified as suppressors or activators of metastatic progression. MicroRNAs can either modulate oncogenic or tumor suppressor pathways or their expression can be regulated by oncogenes or tumor suppressor genes [15]. Studies have shown that miRNAs have critical role in breast cancer including cell proliferation, angiogenesis, invasion and metastasis. Breast cancer metastasis is usually associated with downregulation of antimetastatic miRNA or upregulation of prometastatic miRNA [16].

Till date, antiangiogenic agents available are Bevacizumab (Colorectal cancer), Sunitinib (Renal cell carcinoma, Gastro-intestinal stromal tumours), Sorafenib (Renal cell carcinoma) and metronomic chemotherapy (Breast cancer) [17, 18]. US Food and Drug Administration (FDA) has approved Ramucirumab (Cyramza) that blocks the binding of vascular endothelial growth factor (VEGF) to its receptor VEGFR2 and is used for the treatment of patients with advanced or metastatic, gastric or gastroesophageal junction (GEJ) adenocarcinoma.

Recently, researchers have shown that compounds belonging to isoflavanoid family such as 6-methoxy equol, isoflavanoid genistein, isoflavene-propanol, formononetin [19, 20] and tubulin binding compounds such as TR-644 [21], C-9 [22], β-lactam CA-4 [23] and Azaindole [24] have antivasculature and antimetastatic properties under *in vitro* conditions. Earlier studies have shown antiangiogenic properties of Quinazolino linked 4β-amidopodophyllotoxin conjugates on breast cancer, but a detailed study at molecular level was lacking [25]. Therefore, in the present study, we have investigated the mechanistic aspects of Etoposide and its analogue, Quinazolino-4β-amidopodophyllotoxin (C-10) and how it modulates expression of microRNAs associated with angiogenesis and regulates cell proliferation.

## Materials and Methods

### Cell culture

Human breast carcinoma cell lines MCF-7 and MDA-MB-231 were obtained from American Type Culture Collection (ATCC) and maintained in Dulbecco’s Modified Eagle’s Medium (DMEM) (Sigma-Aldrich), supplemented with 2 mM Glutamax (Invitrogen), 10% fetal bovine serum (Invitrogen), 100 U/ml Penicillin and 100 mg/ml Streptomycin sulfate (Invitrogen). Human Umbilical Vein Endothelial Cells (HUVEC) (Lonza) were maintained in Endothelial Cell Medium (ECM) supplemented with 5% FBS, 100U/ml penicillin, 100 mg/ml streptomycin and 1% Endothelial Cell Growth Supplement (ECGS). All the cell lines were maintained at 37°C in a humidified atmosphere containing 5% CO_2_ in the incubator.

### Determination of cell viability by trypan blue assay

Cell viability measurement in two breast cancer cell lines MCF-7 and MDA-MB-231 treated with Cisplatin, Etoposide or C-10 was studied by trypan blue exclusion assay. In this assay, cells were plated at the density of 5 x 10^4^ cells/ well in 24-well plate and cultured for 24 h followed by treatment with the compounds at 1–16 μM (Etoposide and C-10) and 5–40 μM (Cisplatin) for 24 h. Further, cells were harvested using Trypsin-EDTA (Sigma-Aldrich) and stained with 0.2% Trypan blue (Invitrogen) diluted in DPBS (Sigma-Aldrich). Viable (unstained) as well as dead (stained) cells were counted under the microscope using haemocytometer. The percentage of cell viability was calculated as: Percentage of viability = (Number of unstained cells / Total number of cells) x 100.

### Tube formation assay

A 48-well plate coated with EC matrix and solidified at 37°C was taken and 2 x 10^4^ HUVEC cells (Human umbilical vein endothelial cells) were seeded in each well containing 300 μL of endothelial growth media and allowed to grow for 6 h. Cisplatin, Etoposide or C-10 were added to cells and incubated for 24 h. Then the cells were observed under inverted microscope (Model-CKX41, Olympus) to analyse the tube formation and images were obtained at 4x magnification.

### Wound healing assay (Scratch assay)

MCF-7 and MDA-MB-231 cells were counted and seeded in equal numbers in 12-well plates separately and allowed to grow until it reaches 90% confluence. Then, a scratch was made vertically by using a 200 μL pipette tip; cells were washed with DPBS and supplemented with fresh media. At this time point (0 h), images were captured under inverted microscope (Model-CKX41, Olympus) at 4X magnification. Immediately, cells were treated with compounds Cisplatin, Etoposide or C-10 and incubated for 48 h. Images were obtained at two different time points i.e. at 24 h and 48 h of treatment. All the images taken at three different time points were analysed using CapturePro software (Jenoptik AG).

### Cell cycle analysis

5 x 10^5^ MCF-7 and MDA-MB-231 cells were seeded in 60 mm culture dish and allowed to grow for 24 h. Cells were transfected with 2 μg of pre-microRNA molecules of miR-15, miR-16, miR-17 and miR-221 (PMIRHXXX-PA-1, System Biosciences) cloned in a lentiviral based vector. Further, compounds Cisplatin, Etoposide or C-10 were added to the culture media and cells were incubated for an additional 24 h. Cells were harvested with Trypsin-EDTA, fixed in pre-chilled 70% ethanol at 4°C for 30 min, washed with DPBS and incubated with 1 mg/mL RNase A solution (Invitrogen) at 37°C for 30 min. Cells were collected by centrifugation at 2000 rpm for 5 min, stained with 500 μL of DNA staining solution (10 mg of Propidium Iodide (PI), 0.1 mg of tri-sodium citrate and 0.03 mL of Triton X-100 were dissolved in 100 mL of sterile MilliQ water) and incubated at room temperature for 30 min in the dark. Next, cells were analysed to measure the apoptotic death. DNA contents of 20,000 events were measured by flow cytometer (DakoCytomation, Beckman Coulter, USA) and histograms were prepared using FCS Express Software (De Novo Software).

### MicroRNA expression study

Total RNA was isolated from control (DMSO treated) and compound treated breast cancer cells. An equal amount of DNase-treated RNA was Poly-A tailed using Poly (A) Polymerase and oligo dT adapter to synthesise the cDNA. RT-PCR reaction was set up using universal reverse primer and miRNA specific forward primer. The temperature conditions were 95°C for 10 min followed by 30 cycles of 95°C for 15 sec and 60°C for 1 min. The primers used were obtained from Cancer MicroRNA qPCR array with quanti Mir ^TM^ kit (RA-610A-1, System Biosciences).

### Western blot analysis

Total cell protein from MCF-7 and MDA-MB-231 cells treated with C-10 and positive controls (Etoposide and cisplatin) was isolated by lysing the cells in pre-chilled RIPA buffer (Sigma). After centrifugation at 12,000 rpm for 10 min, the protein in supernatant was collected and quantified by Bradford method (BIO-RAD) using Multimode reader (Varioskan Flash, Thermo-Fischer Scientific). Fifty micrograms of protein per lane was loaded in 12% Sodium Dodecyl Sulfate-Polyacrylamide Gel (SDS-PAGE). After electrophoresis, protein was transferred to polyvinylidine difluoride (PVDF) membrane (Immobilon-P, Millipore). The membrane was blocked at room temperature for 2 h in TBS + 0.1% Tween20 (1X TBST) containing 5% non-fat dry milk blocking powder (Santacruz Biotechnology) followed by washes with 1X TBST for 5 min after which primary antibody was added and incubated at 4°C overnight. Anti-VEGF (IMG-80214) and anti-GAPDH (IMG-6665A) antibodies were purchased from Imgenex Bio., India. Anti-STAT3 (9132L), anti-Bad (9292), anti-Fas (8023) and anti-Bcl-xL (2762) antibodies were obtained from Cell signaling Technology. Anti-Dicer1 (04–721) and anti-Ago1 (07–599) from Millipore; anti-Bcl2 (ab32124), anti-Drosha (ab12286) and anti-TRBP (ab42018) antibodies were obtained from Abcam. Further, membrane was washed and incubated with corresponding horseradish peroxidase-labeled (HRP) secondary antibody (Santacruz Biotechnology) at room temperature for 1h. Membranes were washed three times for 15 min with 1X TBST and visualized with luminol reagent (Luminata Crescendo, Millipore) under ChemiDoc XRS^+^ system (BIO-RAD) and images were captured using Image Lab software.

### Immunoprecipitation (IP)

MCF-7 and MDA-MB-231 cells were treated with C-10 molecule alone and in combination with microRNAs-15, 16, 17 and 221 separately and incubated for 24 h. Total protein was isolated from control and treated cells with NP40 lysis buffer. The protein lysate was incubated with anti-STAT3 antibody (9132L, CST) and anti-IgG overnight at 4°C. The above mixture was further incubated with magnetic beads (Clontech at 4°C for 2 h. The beads were washed three times with wash buffer containing protease inhibitor and resuspended in 5X loading dye followed by heating at 90°C for 5 min. Later these samples were electrophoresed on SDS-PAGE and western blotting was performed.

### Caspase-9 assay

Caspases-9 assay was performed according to the manufacturer’s recommendations using Apoalert caspase-9/6 fluorescent assay kit (Clontech, CA, USA). MCF-7 and MDA-MB-231 cells were transfected with microRNA clones (miR-15, 16, 17 and 221) followed by treatment with Cisplatin, Etoposide or C-10 and incubated for 24 h. Total protein was isolated from cells harvested using Trypsin-EDTA. Equal amount of protein was loaded in each well of 96 well plate and the substrate (LEHD–AMC) was added to the cell lysates and incubated for 1 h at 37°C. Readings were taken at λ excitation 400 nm and λ emission 505 nm in multimode reader (Varioskan Flash, Thermo Scientific).

### Molecular modelling studies

To dock C-10 and Etoposide at SH2 domain of STAT3, AutoDock was employed [26]. Initial Cartesian coordinates for the protein-ligand complex structure were derived from crystal structure of STAT3 (PDB ID: 1BG1). The protein targets were prepared for molecular docking simulation by removing water molecules and bound ligands. Hydrogen atoms and Kollman charges were added to each protein atom. Auto-Dock Tools (ADT) was used to prepare and analyze the docking simulations for the AutoDock program. Coordinates of each compound were generated using Chemdraw11 followed by MM2 energy minimization. Grid map in Autodock that defines the interaction of protein and ligands in binding pocket was defined. Grid box size of 80 x 80 x 80 Å was selected that include the whole SH2 dimerization domain of STAT3 monomer. AutoGrid 4 was used to produce grid maps for AutoDock calculations where the search space size utilized grid points of 0.375 Å. The Lamarckian genetic algorithm was chosen to search for the best conformers. Each docking experiment was performed 100 times, yielding 100 docked conformations. Parameters used for the docking were as follows: population size of 150; random starting position and conformation; maximal mutation of 2 Å in translation and 50 degrees in rotations; elitism of 1; mutation rate of 0.02 and crossover rate of 0.8; and local search rate of 0.06. Simulations were performed with a maximum of 1.5 million energy evaluations and a maximum of 50000 generations. Final docked conformations were clustered using a tolerance of 1.0 Å root mean square deviation. The best model was picked based on the best stabilization energy. Final images for molecular docking were conveniently generated by using PyMol programme [27].

### Transfection studies

STAT3 protein coding Adenoviral based expression vector pAdSTAT3, control vector pAdtrack-CMV and shRNA plasmid targeting STAT3 were kind gift from Dr. Nishant Jain (Indian Institute of Chemical Technology, India). Adenoviral vector pAdSTAT3 was generated by the insertion of STAT3 cDNA into AdEasy^TM^ adenoviral vector system (Stratagene). The inserted STAT3 cDNA was under the control of Cytomegalovirus (CMV) promoter and terminated by Simian Virus 40 (SV40) polyadenylation signal. This plasmid constructs co-expresses green fluorescent protein (GFP) to monitor infection efficiency [28]. VEGF Promoter sequences P1 [spanning region of -946/+100 containing STAT3 (-848 bp) binding site] and P2 [spanning region of -1262/+100 has Sp1(-1100 bp), HIF-1α (-951 bp, -978 bp) and STAT3 (-848 bp) binding sites] were cloned in pGL3 basic vector containing luciferase reporter gene and transfected at a concentration of 2 μg along with pCMV-β gal (0.5 μg) in MCF-7 and MDA-MB-231 cells separately for 24 h. This was followed by treatment with Cisplatin, Etoposide or C-10 for 24 h. In recovery experiment, cells were treated with C-10 and transfected with VEGF clones followed by STAT3 overexpression with pAdSTAT3 viral infection. The protein lysates were subjected to luciferase reporter assay. The luciferase gene expression is the indirect measure of promoter activity. The primers used for P1 clone (-946/+100) are

Forward: 5’-ATGCAAGCTTCTGCCGCTCACTTTGATGT-3’;

Reverse: 5’-GATCGCGGCCGCCGCTACCAGCCGACTTTT-3’ and

P2 clone (-1262/+100) are

Forward: 5’-ATGCAAGCTTGAGACGAAACCCCCATTTCT-3’; and

Reverse: 5’-GATCGCGGCCGCCGCTACCAGCCGACTTTT-3’.

The restriction enzyme sites used for cloning of VEGF promoter sequences are HindIII and NotI. In this study, CMV-β galactosidase plasmid was used for normalizing the transfection efficiency.

### Statistical analysis

Statistical analysis was performed using the graph-pad software to evaluate the significant difference between the control and treated samples. All variables were tested in three independent experiments. The results were reported as mean ± SD. * represents p-value < 0.05, ** represents p-value < 0.01 and *** represents p-value < 0.001.

## Results

### Quinazolino-4β-amidopodophyllotoxin (C-10) affects cell viability, angiogenesis and cell migration

**Fig 1:**
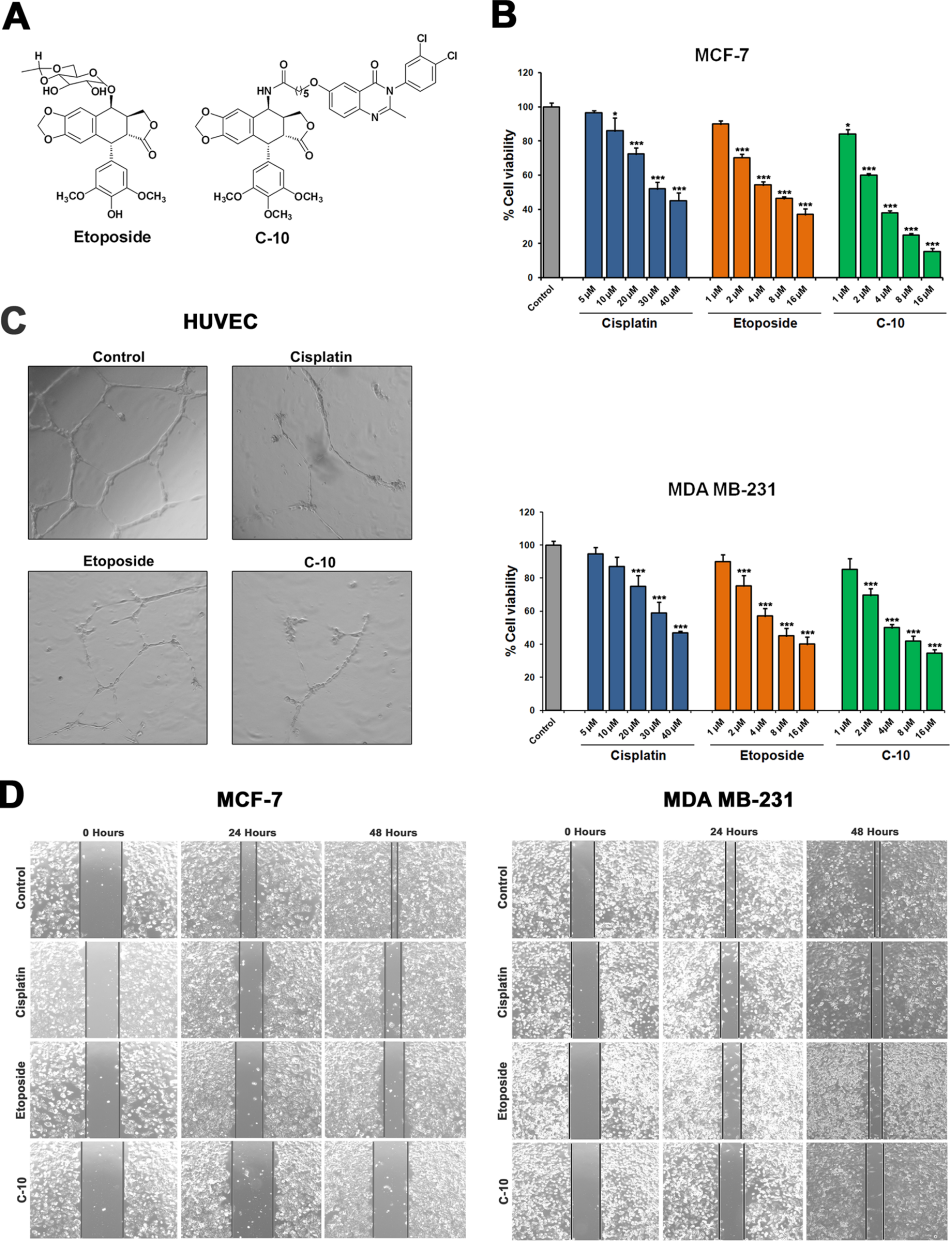
Chemical structure and anti-cancer activities of Etoposide and C-10 on breast cancer cells. (A) Chemical structure of Etoposide and C-10 [4β-[6-(3, 4-dichlorophenyl) 3, 4-dihydro-2-methyl-4-oxoquinazolin-6-yloxy) hexanamide]-4-desoxy-podophyllotoxin]. (B) Trypan blue assay for cell viability on MCF-7 and MDA-MB-231 cells showing gradual decrease in viability after treatment with Etoposide or C-10 at 1–16 μM or Cisplatin at 5–40 μM and incubated for 24 h. (C) HUVEC cells were grown on EGM media and treated with Cisplatin, Etoposide or C-10 compounds after tube formation. Drastic inhibitory effect was observed upon C-10 treatment. (D) Wound healing assay on MCF-7 and MDA-MB-231 cells showing antiangiogenic and antimigratory effects after treatment with C-10 for 48 h. Images were obtained at 0 h, 24 h and 48 h by using an inverted microscope with 4X objective lens.

We measured the toxic effect of Quinazolino-4β-amidopodophyllotoxin (C-10) on cell viability in MCF-7 and MDA-MB-231 breast cancer cell lines. Trypan blue assay based on the principle that live cells with intact cell membrane excludes the trypan blue, whereas dead cells do not was performed. The MCF-7 and MDA-MB-231 cells were treated with Etoposide or C-10 at 1–16 μM and Cisplatin at 5–40 μM and incubated for 24 h. There was a 50% reduction in cell viability when treated with Etoposide or C-10 at 4 μM concentration and Cisplatin at 30 μM (Fig 1B). Previous studies with deoxy-podophyllotoxin conjugates have shown inhibitory activity on angiogenic tube formation in HUVEC cells and exhibited cytotoxic effects in A549, SK-OV-3, SK-MEL-2, HCT-15 and B16F10 cancer cells [29, 30]. Thus, we quantified the potential inhibitory activity of C-10 on angiogenesis by conducting *in vitro* angiogenic assay. HUVEC cells were seeded on matrigel and allowed to form capillary tube like network for 6 h followed by treatment with Cisplatin, Etoposide or C-10. Images were observed under microscope and analyzed. Interestingly, C-10 treated HUVEC cells resulted in drastic decrease in the percentage of tube formation compared to Cisplatin and Etoposide confirming its effective role in angiogenesis (Fig 1C).

Further, we were interested to find out antimigratory effect of C-10. Therefore, we performed wound healing assay in both MCF-7 and MDA-MB-231 cells separately by making a scratch in the middle of the each well of a 12-well plate. The MCF-7 and MDA-MB-231 cells were grown until it reached 90% confluence. Thereafter, a scratch was made vertically in the middle of the well by using 200 μL pipette tip. Cells were treated with compounds and incubated for 48 h. Images were obtained at 0 h, 24 h and 48 h by using an inverted microscope with 4X objective lens. Interestingly, cells treated with C-10 showed less migratory effect compared to Cisplatin and Etoposide (Fig 1D). Based on these observations it is clear that C-10 showed a significant reduction in cell viability and also showed antiangiogenic and antimigratory effects compared to positive controls.

### C-10 modulates the expression of microRNAs involved in angiogenesis

**Fig 2:**
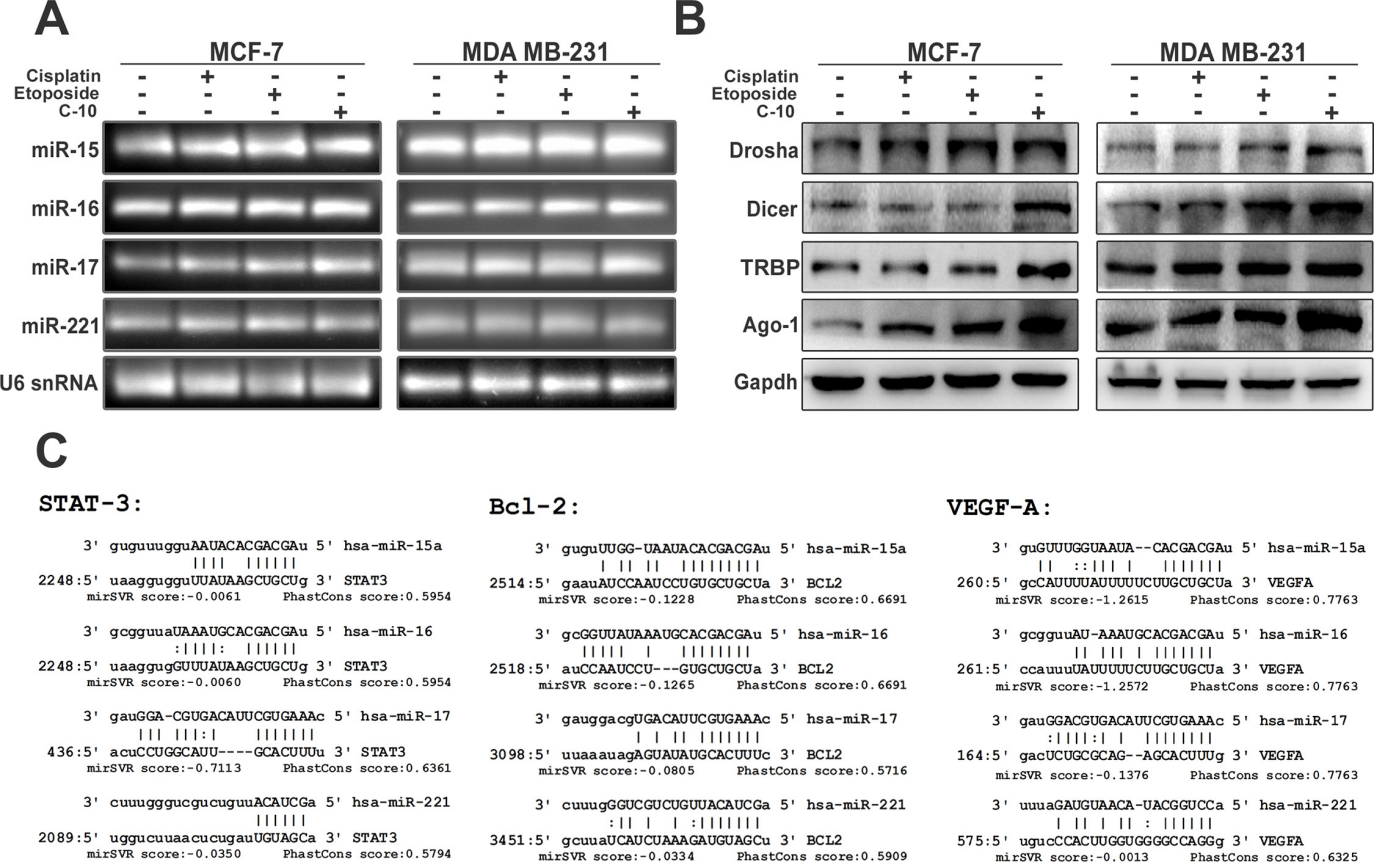
C-10 modulates microRNA expression and its biogenesis. (A) Endogenous microRNA expression studies in compound treated MCF-7 and MDA-MB-231 cells showing significant upregulation of miR-15 and miR-16 in Etoposide or C-10 treated cells compared to miR-17 and miR-221. (B) The C-10 compound enhanced the expression of Drosha, Dicer, TRBP and Ago-1 enzymes that involved in synthesis and processing of matured microRNAs. (C) Computational analysis of miRNA prediction shows the possible binding sites in 3’UTR of Bcl-2, STAT3 and VEGFA for each miRNA-15, 16, 17 and 221 along with miSVR scores as depicted by miRanda software.

Recently, microRNAs have emerged as new promising players that control expression of genes involved in various cellular processes. miRNAs regulate angiogenesis by changing the expression of pro and antiangiogenic factors and endothelial cell function. Aberrant expression of miR-15a/16 causes decrease in the expression of VEGF-A and play a vital role in tumorigenesis particularly in Multiple Myeloma (MM) [31–34]. Studies suggest that ectopic expression of miR-17 and miR-221 inhibit cell proliferation and migration by targeting STAT3 and induce apoptosis [35, 36]. In another study, it was evidenced that the Quinazoline based small molecules enhance the global upregulation of microRNAs involved in the process of apoptosis in breast cancer cells [37]. Therefore, we studied the expression pattern of these miRNAs in MCF-7 and MDA-MB-231 breast cancer cell lines. Cells treated with Cisplatin, Etoposide or C-10 were incubated for 24 hours and subjected to endogenous miRNA expression studies. Interestingly, we observed that both Etoposide and C-10 induced significant increase in expression of miRNAs-15 and 16 and also there was a modest increase in expression of miR-17 and 221 (Fig 2A). Studies by different groups reported that small molecules enhance RNA interference and promote microRNA processing by enhancing the expression of TRBP, which is an integral component of Dicer1 complex that plays a critical role in microRNA processing [38, 39]. Therefore, to understand the mechanism underlying the upregulation of these microRNAs, we investigated whether C-10 has any regulatory potential role on expression of microRNA processing enzymes Drosha, Dicer, TRBP and Ago-1 that play a major role in the microRNA biogenesis pathway. We observed that C-10 molecule positively regulate both nuclear microprocessor enzyme Drosha and the cytoplasmic Dicer, TRBP and Ago-1 in MCF-7 and MDA-MB-231 cells (Fig 2B). These observations clearly suggested that C-10 molecule modulated the expression of microRNAs-15, 16, 17 and 221 by inducing microRNA processing machinery.

### C-10 induces apoptosis in combination with microRNAs

**Fig 3:**
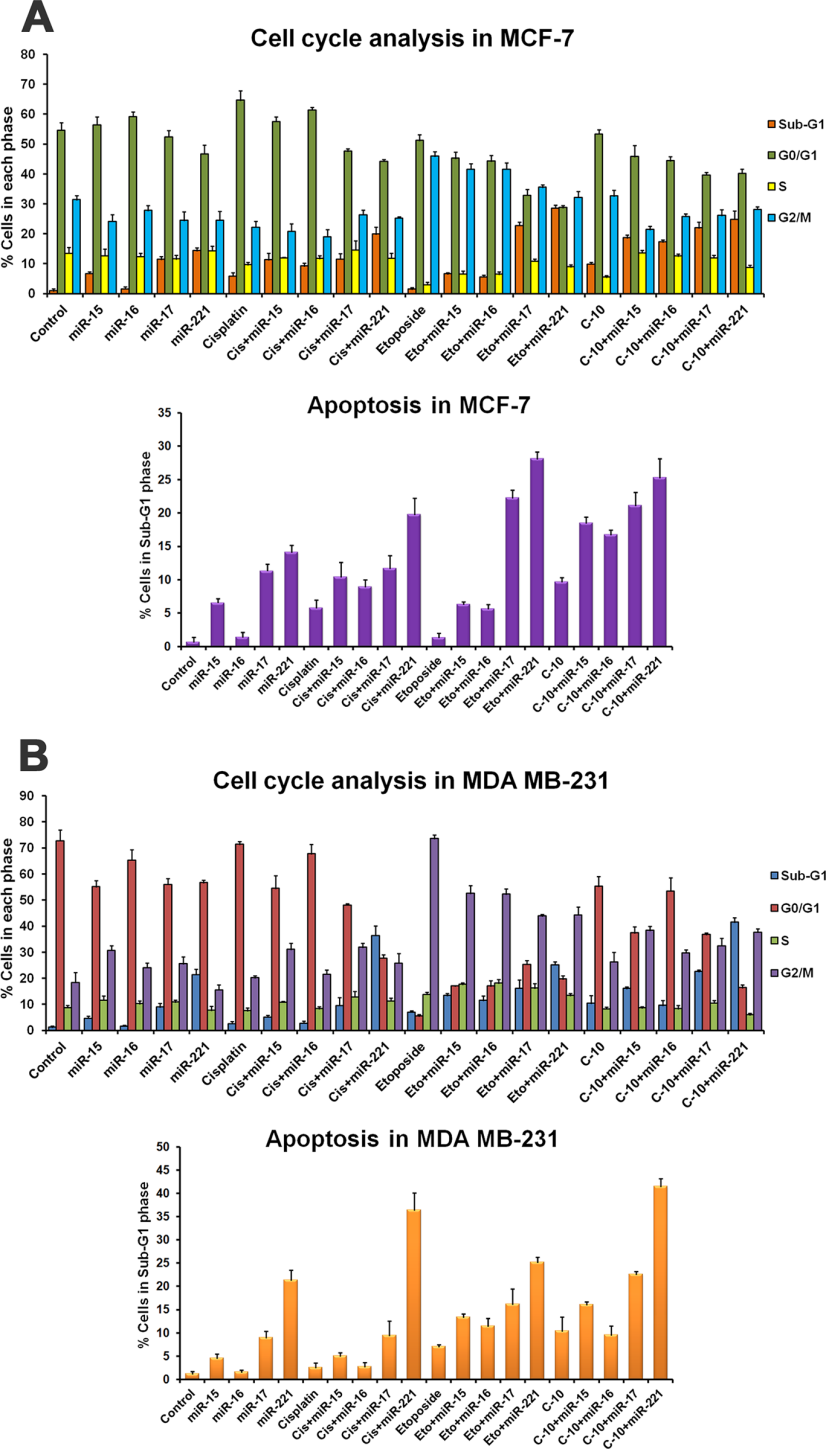
Combinatorial effect of C-10 and microRNAs on cell cycle and apoptosis. (A and B) MCF-7 and MDA-MB-231 cells transfected with 2 μg each of precursor microRNAs (miR-15, 16, 17 and 221) for 24 h followed by treatment with Etoposide or C-10 at 4 μM concentration for 24 h. FACS analysis data showed an increase in percentage of apoptosis, in combination of microRNA and C-10.

miR-15 and miR-16 act as putative tumor suppressors and target the genes such as Bcl-2 and VEGF that are implicated in regulation of cell cycle, apoptosis and proliferation [34, 40, 41]. In a previous study, it was demonstrated that miR-17 targets STAT3 in tumor microenvironment in melanoma mouse model and it was also supported by computational studies which showed STAT3 as a putative target of miR-17 [35]. In addition, miR-221 was found to be overexpressed in various cancers and is tightly associated with VEGF dependent signalling pathway. When enormous miR-221 is provided exogenously, it prevents cell proliferation and migration in endothelial cells [36]. Based on the alignment studies with microRNA prediction tools like microrna.org (miRanda), TargetScan, PITA and RNAhybrid we found that miR-15, 16, 17 and miR-221 could efficiently target the genes like Bcl-2, STAT3 and VEGF which are the key players of apoptosis and angiogenesis (Fig 2C and S4 Fig). Therefore, we examined the ectopic expression of these miRNAs in inducing apoptosis. MCF-7 and MDA-MB-231 cells were transfecd with microRNA over expressing plasmid DNA (miR-15, 16, 17 and 221) for 24 h followed by compound treatment. FACS analysis was performed to see the effect on cell cycle. Results indicated an increase in apoptosis in cells transfected with miR-15, 16, 17 and 221 compared to treatment with C-10 compound alone. Interestingly, the combination of microRNA and compound C-10 showed an enhancement in the percentage of apoptotic cells in a synergistic manner (Fig 3 and S2 Fig).

### miR-15, 16, 17 and 221 control cell proliferation and angiogenesis by targeting STAT3

**Fig 4:**
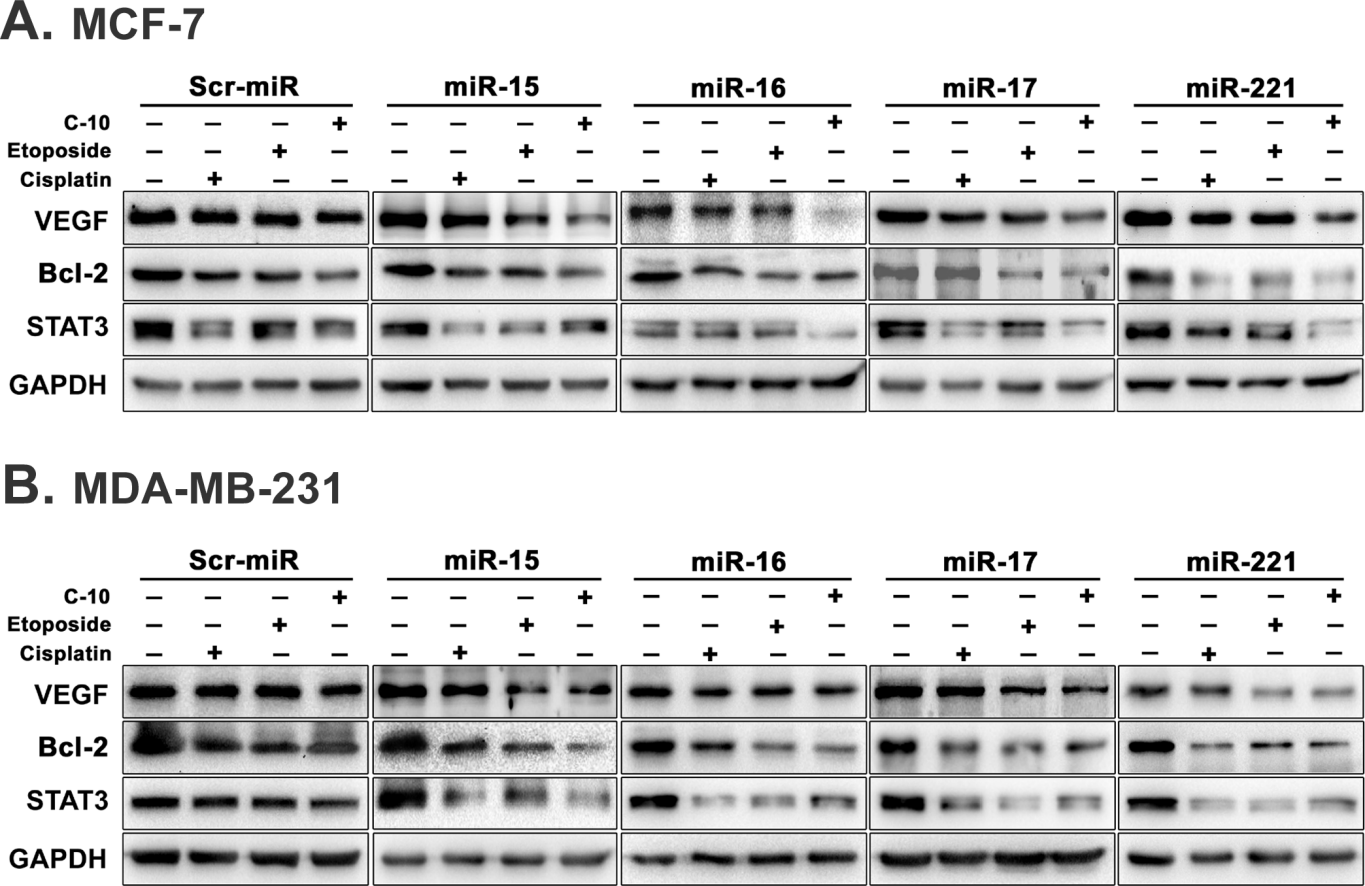
Combinatorial effect of C-10 and microRNAs on its target genes. (A and B) MCF-7 and MDA-MB-231 cells were transfected with microRNA-15, 16, 17 and 221 for 24 h followed by treatment with Cisplatin, Etoposide or C-10 and incubated for 24 h. Western blot analysis showing decrease in levels of Bcl-2, STAT3 and VEGF proteins. GAPDH was used as loading control in all combinations separately.

MicroRNAs regulate gene expression by binding to 3’UTR of the target mRNA. Growing evidences have shown that miRNAs associated with STAT signalling pathway play regulatory role in STAT3 mediated tumorigenesis. Moreover, computational studies also have shown STAT3 as a putative target of microRNAs-15, 16, 17 and 221. Recent studies have shown that STAT3 is thought to promote oncogenesis by regulating several genes required for cell survivial (Bcl-2, Bcl-xL, Mcl-1, Survivin), proliferation (Cyclin D1, c-Myc) and angiogenesis (VEGF) [42]. In breast cancer cells, it is reported that VEGF acts as survival factor and prevents apoptosis by inducing Bcl-2 expression [43, 44]. In another study, it is showed that Bcl-2 induces VEGF expression in neovascular endothelial cells through STAT3 mediated pathway [45]. In addition studies provide evidence that Bcl-2 and VEGF genes are regulated directly by STAT3 protein which clearly suggest that STAT3 represents a common molecular target to control cell proliferation and angiogenesis and is considered as an oncogene [46–48]. Hence, we examined the effect of these microRNAs over STAT3 and its putatibe targets Bcl-2 and VEGF. The MCF-7 and MDA-MB-231 cells werer transfected with each of these microRNA constructs (miR-15, 16, 17 and 221) followed by treatment with Cisplatin, Etoposide or C-10 for 24 h. Total protein was isolated and western blot analysis was performed. A significant decrease in the expression of all the three targets was observed in cells transfected by microRNA and treated with C-10 compound in comparison to untransfected and treated. Further, we observed a significant decrease in expression of antiapoptotic protein Bcl-xL and increase in the poapoptotic protein Bad in C-10 treated MCF-7 and MDA-MB-231 cells that suggest the activation of intrinsic pathway of apoptosis. We also found no change in the expression of Fas protein which gives the evidence for quiescence of extrinsic apoptosis pathway (S3 Fig). These findings clearly suggest that combination of miRNAs with the compound C-10 resulted in drastic reduction in expression of target proteins when compared to compound treatment alone (Fig 4).

### Combinatorial treatment of C-10 and microRNA activates Caspase-9

**Fig 5:**
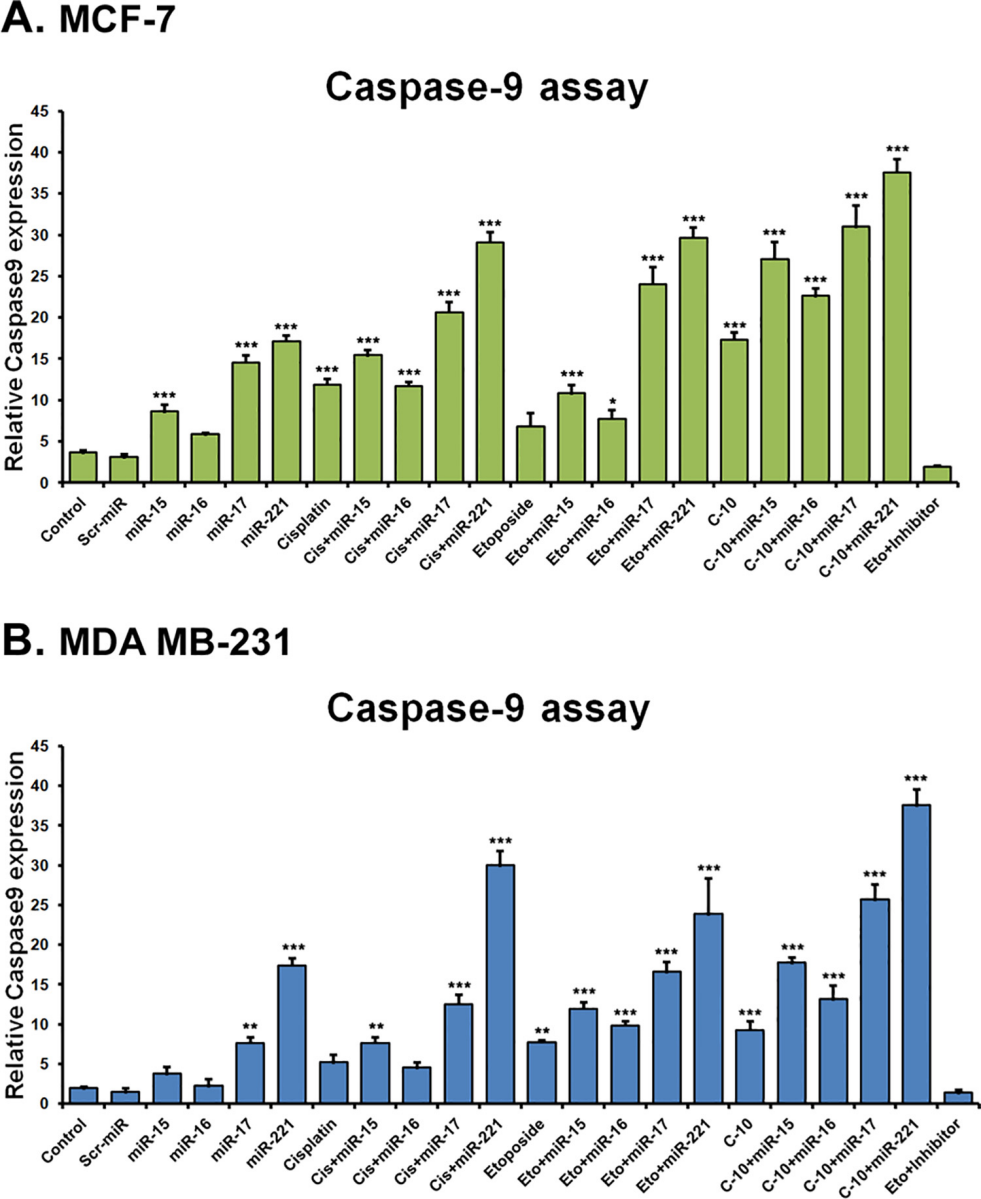
Caspase-9 assay. (A and B) Apoalert Caspase-9 fluorescent assay showing the apoptotic inducing ability of Etoposide or C-10 separately and in combination with miR-15, 16, 17 and 221. Results represent mean ± SD of three independent experiments. * represents p-value < 0.05, ** represents p-value < 0.01 and *** represents p-value < 0.001.

Apoptosis is an essential regulatory process that maintains homeostasis in multicellular organisms by balance of antiapoptotic and proapoptotic proteins. A large scale screening of complete miRNA mimic library demonstrated that miR-15 and miR-16 activate caspase-3, 8 and 9 by targeting Bcl-2 in rat hepatic stellate cells [49–51]. Further, it is confirmed that, when MEG-01 cells were transfected with miR-15 and miR-16 an increased rate of apoptosis mediated by the cleavage of procaspase-9 and PARP was observed. This study suggests that targeting Bcl-2 by these miRNAs alone is adequate to initiate apoptosis [52]. Recently, it is found out that Bcl-2 is the therapeutic target of miR-17-92 cluster in BCR-ABL positive Acute Lymphoblastic Leukemia (ALL) [53] and overexpression of miR-221/222 downregulate Bcl-2 and induce apoptosis in gastrointestinal stromal tumors [54]. Since, the compound C-10 modulated the expression of miR-15, 16, 17 and 221 and caused apoptosis, we analysed its effect on caspase-9 activity by performing Apoalert Caspase-9 fluorescent assay in MCF-7 and MDA-MB-231. We observed a significant increase in caspase-9 activity which was further enhanced in cells transfected with miR-15, 16, 17 and 221 (Fig 5).

### C-10 inhibits STAT3 protein by direct binding

In order to understand the role of C-10 on STAT3, we performed the molecular modeling studies for C-10 in comparison with Etoposide against the STAT3 SH2 domain. Autodock4 was employed to carry out the docking experiments and proposed images were generated by PyMol software. The compound C-10 showed successful docking simulations at the SH2 domain and the crystal structure of STAT3 (PDB code: 1BG1) was retrived from RSCB protein data bank [26, 27, 55]. Based on review of literature it is well Affirmed that the SH2 domain accomodated by amino acid residues like Glu612, Glu530, Lys591, Tyr575, Arg595, Ser613, Gly618, Ser636, Val637, Arg609, Thr620, Glu638, Pro639 and Ile634. The compound C-10 mainly consist of three structural appendages like azapodophyllotoxin, dichlorophenyl quinozolin and carbon chain as a linker. However, azapodophyllotoxin segment surrounded by Gln635, Ser639, Ser636, Glu638, Pro639 and a strong hydrogen bonding interaction was observed between aza NH atom and carbonyl O atom of Ser636 (NH—-O, distance = 2.0 Å). Interestingly the NH atom of Gln635 forms two hydrogen bonds with O atom of lactom ring as well as carbonyl O with bonding distances of 2.9 and 3.1 Å respectively (O—-NH—-O). In addition the dichlorophenyl quinozolin moiety with polar ring N atom showed hydrogen bonding with carbonyl O of Lys591 (NH—-O, distance = 2.7 Å). Moreover, the carbon chain placed between these two segments exhibited hydrophobic interactions with the amino acid residues like Tyr575, Gly618, Pro639 and Leu571.

**Fig 6:**
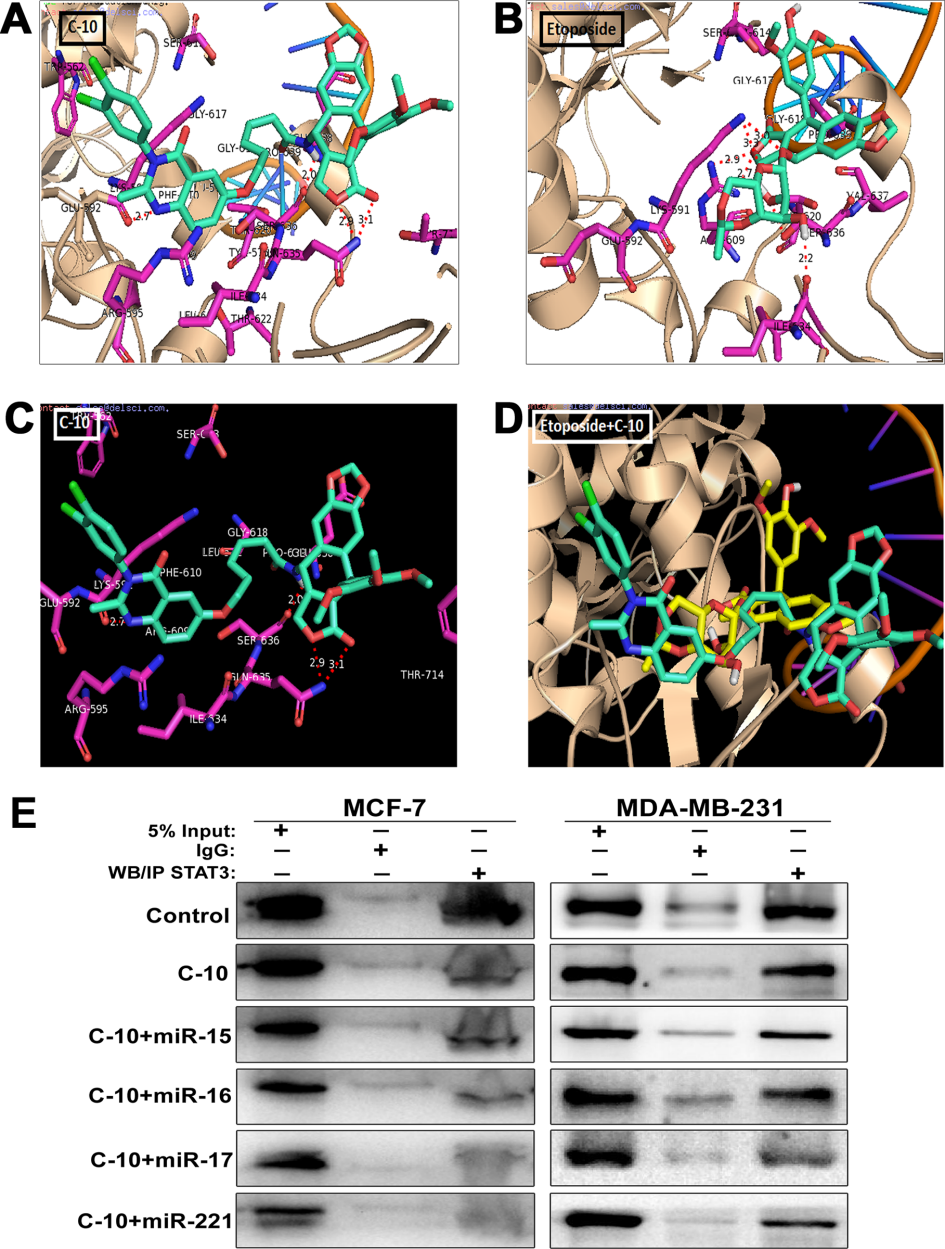
C-10 physically interacts with STAT3. (A and B) The panel of poses represent the molecular docking interactions of C-10 and Etoposide ligands on STAT3 at SH2 pocket. The backbone of protein has been shown as *wheat* colored cartoon and the ligands that have docked are dipicted as *green* colored sticks model. The interacting amino acid residues are shown in magenta sticks and potential inter molecular hydrogen bondings were displayed in *red* dashes. (C) The poses with black background indicate C-10 with residue in megenta colored significant residues. (D) Image represents C-10 superimposes on Etoposide (yellow stick) suggestive of the same site in the SH2 domain. PyMol programme has been employed for the visualization of docked images. (E) STAT3 protein was separated from total cell protein by immunoprecipitation method and western blotting was performed to analyze the expression of STAT3. Immunoglobulin-G (IgG) was used as negative control.

The basic compound etoposide consists of podophyllotoxin structure with a glucoside unit and absence of carbon chain linker as well as quinozolin structral units. The O atom of lactom ring forms two hydrogen bonds with two NH atoms of Arg609 with bonding distances of 2.7 and 2.9 Å (NH—-O—-HN). In addition, weak hydrogen bonding was observed between NH of Lys591 O atoms of podophyllotoxin as well as glucosidic unit (O—-NH—-O). Further we noticed a strong hydrogen bonding interaction between carbonyl O atom of Ile634 and H atom of hydroxyl unit of glucoside (OH—-O distance = 2.2 Å), additionally another hydroxy O atom of same ring forms hydrogen bonding interaction with OH of Ser636 (O—-OH distance = 3.0 Å). However, a wide range of molecular docking interaction were found to be absent in the case of etoposide due to unavailability of carbon chain linker and quinozolin structural units when compared to C-10. For better clarification of this a superimposition of both compounds was analyzed. The observations were in accordance with the experimental results; in particular, dichlorophenyl quinozolin moiety placed in a tight hydrophobic region at SH2 domain and this could be a critical unit for C-10 molecule to show successful docking interactions at the site.(Fig 6A–6D).

Based on the molecular docking studies we were interested to know the binding ability of C-10 molecule with STAT3 protein when C-10 combined with microRNAs-15, 16, 17 and 221. We performed Immunoprecipitation assay (IP) to separate STAT3 from total protein to see whether any change existing in the expression of STAT3. This assay was performed in cells treated with C-10 and microRNAs in combination. Interestingly, we observed a significant decrease in expression of STAT3 in MCF-7 and MDA-MB-231 cells treated with C-10 and the expression was further decreased in combination treatment achieved with microRNAs-15, 16, 17 and 221. These results clearly suggested that the binding ability of C-10 with STAT3 protein remains unchanged though the microRNAs were involved in combination therapy (Fig 6E).

### Role of C-10 on VEGF promoter activity

**Fig 7:**
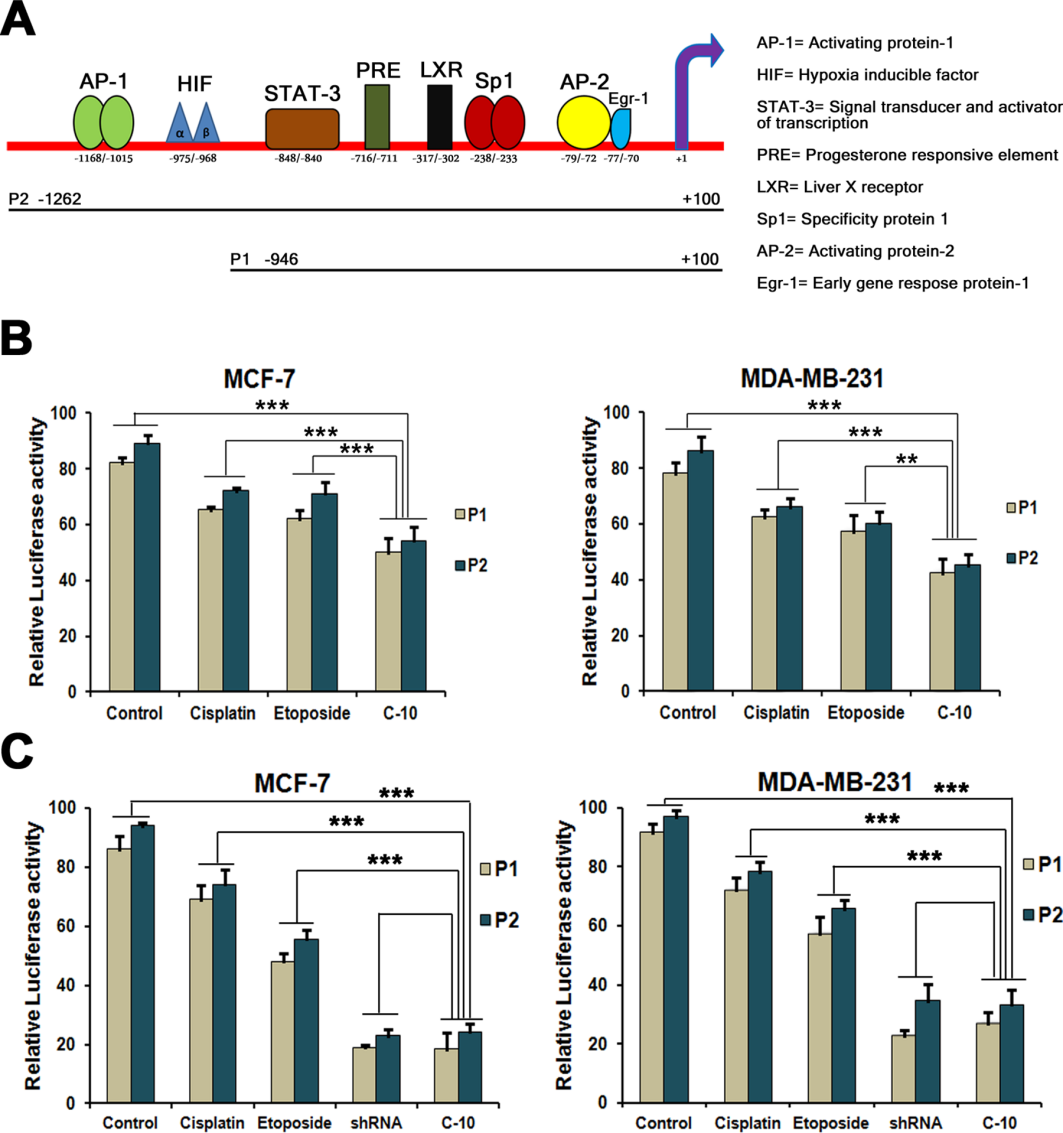
Effect of C-10 on VEGF promoter activity. (A) Schematic representation of VEGF promoter with binding sites for different transcription factors. (B) The VEGF promoter activity was monitored by measuring the Luciferase expression in MCF-7 and MDA-MB-231 cells transfected with VEGF promoter sequences followed by compound treatment. In this assay, pCMV-β gal was used to normalize the transfection efficiency. (C) The recovery experiment was performed to prove STAT3 dependent VEGF promoter activity. The cells were treated with compounds and transfected with VEGF promoter sequences followed by STAT3 overexpression by pAdSTAT3 vector. Simultaneously, STAT3 shRNA was transfected to knockdown the STAT3 expression. Results represent mean ± SD of three independent experiments. * represents p-value < 0.05, ** represents p-value < 0.01 and *** represents p-value < 0.001.

In estrogen positive breast cancer cells, estrogen receptors α and β have a direct transcriptional effect on VEGF gene [56, 57]. Moreover, STAT3 as a transcription factor regulates transcriptional activity of VEGF gene by binding to VEGF promoter at -848 bp region [47]. It has been shown that Quinazolino linked 4β-amidopodophyllotoxin conjugates regulate angiogenesis by VEGF dependent pathway [25]. Since, the compound C-10 downregulated STAT3 activity, we were interested to study the transcriptional regulation of VEGF promoter upon treatment with Etoposide or C-10. We cloned VEGF promoter sequences in pGL3 basic vector containing luciferase reporter gene and also used CMV-β gal plasmid as internal control for transfection efficiency. VEGF promoter clones [P1 spanning region of -946/+100 containing STAT3 (-848) binding site and P2 spanning region of -1262/+100 has Sp1 (-1100), HIF-1α (-951, -978) and STAT3 (-848) binding sites] [58] were transfected in MCF-7 and MDA-MB-231 cells separately for 24 h followed by treatment with Cisplatin, Etoposide or C-10 and incubated for another 24 h. A drastic reduction in VEGF promoter activity (i.e normalized luciferase values) was observed in cells treated with C-10 compared to Cisplatin or Etoposide (Fig 7B). Based on these results, it is known that the decrease in promoter activity correlated to decreased availability of STAT3 in compound treated cells [47]. The reduction was less pronounced when P2 construct (-1262/+100) was used, possibly due to the strong transcriptional activation mediated by binding of HIF-1α transcription factors that help in recruiting other survival factors [59]. Further, to prove that STAT3 regulates VEGF promoter activity we performed the recovery experiments in which cells were treated with C-10 molecule followed by transfection of VEGF promoter clones (P1 and P2) and overexpressed STAT3 protein with adenoviral vector pAdSTAT3. Simultaneously in a separate experiment we also co-transfected one of the samples with STAT3 shRNA plasmid along with promoter sequences (Fig 7C). We used pAdtrack-CMV as control vector which does not code for STAT3 protein. Luciferase expression was noticed as measure of promoter activity. We observed tremendous increase in STAT3 protein level in samples treated with STAT3 overexpressed clones that was suppressed upon compound treatment compared to the untreated controls. The cells treated with STAT3 shRNA showed a drastic decrease in the promoter activity which represents unavailability of STAT3 protein due knockdown. A similar kind of STAT3 inhibition pattern was also observed upon addition of C-10 molecule. These results evidenced that STAT3 is required for transcriptional activation of VEGF gene which could be effectively suppressed by C-10 molecule.

## Discussion

In our previous studies, we found Etoposide and its analogues (Quinazolino linked 4β-amidopodophyllotoxin conjugates) cause cell cycle arrest and apoptosis, but the molecular mechanism of action was not explored to the full extent. In the present study, we demonstrated that Etoposide and its analogue C-10 exhibit strong antiproliferative, antiangiogenic and apoptosis inducing nature by modulating the expression of microRNAs that are strongly associated with tumor suppression and angiogenesis. Etoposide and C-10 cause decrease in cell viability and triggered apoptosis in MCF-7 and MDA-MB-231 breast cancer cells at 4 μM concentration (Figs 1B and 3). However, increased concentration of compounds resulted in increased apoptosis. Next, we performed *in vitro* angiogenesis assay (tube formation assay) by using HUVEC cells treated with C-10. We observed a drastic reduction in tube length in C-10 treated cells compared to Cisplatin and Etoposide (Fig 1C). Further, to know the antimigratory effect of C-10, we performed wound healing assay/scratch assay by which we confirmed that our compound C-10 also has effective antimigratory nature (Fig 1D).

It is known that microRNAs regulate post transcriptional gene regulation by binding to 3’UTR of several mRNAs [3, 4, 6]. Based on earlier studies by different research groups and microRNA binding site prediction tools (miRanda, TargetScan, PITA and RNAhybrid) we have selected microRNAs-15, 16, 17 and 221 associated with cell proliferation and angiogenesis (Fig 2C). Initial RT-PCR analysis of samples showed a significant upregulation of miR-15, 16, 17 and 221 in MCF-7 and MDA-MB-231 cells treated with Etoposide or C-10 suggesting that C-10 compound has the potentiality to modulate these microRNAs that play a crucial role in angiogenesis and tumor suppression, which in turn leads to an increase in apoptosis (Fig 2A). Further, to understand the mechanism of C-10 mediated microRNA upregulation, we observed the expression of microRNA processing enzymes like Drosha, Dicer, TRBP and Ago-1 in C-10 treated cells. Interestingly, we observed a significant increase in the expression of microRNA processing enzymes that confirms the activity of C-10 molecule which positively regulated the expression of microRNAs-15, 16, 17 and 221 (Fig 2B). This data strongly correlates with earlier studies where in miR-15, miR-16 and miR-17 cause cell cycle arrest and apoptosis by targeting key protein molecules such as VEGF, Bcl-2 and STAT3 [34, 35, 40, 41, 52].

STAT3 signaling plays a major role in the intrinsic pathway of cancer inflammation and participates in oncogenesis through upregulation of genes involved in proliferation, antiapoptosis and angiogenesis [40, 60]. Proteins such as VEGF and STAT3 play crucial roles in angiogenesis [8–10]. Studies showed that binding of miR-15 and miR-16 to mRNA of VEGF control angiogenesis process [33]. In previous studies, we observed Quinazolino linked 4β-amidopodophyllotoxin conjugates induced a decrease in levels of both VEGFA and STAT3 proteins [25]. Thus, we were interested to understand the mechanistic role of C-10 and microRNAs-15, 16, 17 and 221 on STAT3 expression. Interestingly, cells treated with compound C-10 resulted in reduced expression of STAT3 protein which was further enhanced in combinatorial treatment (Fig 4). Recent studies have emphasized that miR-15 and miR-16 induce apoptosis by activating caspases [49]. Therefore, we performed fluorescence based caspase-9 assay to observe the possible involvement of caspase proteins in the activation of apoptosis. Amazingly, a significant increase in caspase-9 activity was observed in MCF-7 and MDA-MB-231 cells treated with C-10, which was further enhanced when microRNAs-15, 16, 17 and 221 were added externally (Fig 5).

Further, docking studies was conducted to elucidate the possible binding modes of Etoposide and its analogue C-10 with STAT3 protein. We found that C-10 exhibited possible bonding at various positions due to its structural variance that has azapodophyllotoxin, dichlorophenyl quinozolin and carbon chain linker. Whereas, Etoposide has same podophyllotoxin structure but lacks quinozolin and carbon chain linker which are crucial for additional bonding. This stuctural difference, specifically the dichlorophenyl quinozolin moiety of C-10 molecule could be a critical unit for successful docking interactions at the site.(Fig 6A–6D). These findings distinctly suggested that C-10 plays a crucial role in the regulation of STAT3 by direct binding. As we mainly focussed on combination therapy with C-10 and microRNAs, we checked the binding ability of C-10 with STAT3 protein by performing Immunoprecipitation followed by western blotting. These results clearly indicated that the binding ability of C-10 remains the same and a drastic decrease in STAT3 expression was observed in combination treatment with C-10 and microRNAs-15, 16, 17 and 221 (Fig 6E)

Reports on angiogenesis showed that VEGF plays crucial role in regulating new blood vessel formation [4] and Etoposide and its conjugates regulate VEGF dependent pathway [25]. Thus, we suspected possible role of compound C-10 on transcription of VEGF gene. VEGF promoter constructs (P1 and P2) were transfected in breast cancer cells followed by compound (Eto and C-10) treatment. We observed a significant decrease in promoter activity in P1 plasmid construct carrying mainly STAT3 binding region. But, the reduction was found to be moderate in case of P2 construct that contains binding sites for HIF-1α and STAT3, possibly due to strong transcriptional activity exerted by HIF-1α (Fig 7). Further, STAT3 dependent transcriptional activity of VEGF gene was proved by recovery experiments done with STAT3 overexpression vector pAdSTAT3 and STAT3 shRNA. A clear decrease in expression of VEGF gene in cells where STAT3 is knocked down by shRNA proved that STAT3 directly regulates VEGF gene expression. Similar kind of effect was observed with C-10 molecule which correlates with result of shRNA. Finally, based on all these findings we conclude that the strong apoptotic and antiangiogenic effect caused by Etoposide and its analogue C-10 in combination with miR-15, 16, 17 and 221 demonstrate a clear evidence for the therapeutic potential of these compounds for treatment of breast cancer.

## Supporting Information

S1 Fig A&B. HPLC pattern of Etoposide and C-10 compounds. (DOCX)

S2 Fig A&B. Combinatorial effect of C-10 and microRNAs on cell cycle. MCF-7 and MDA-MB-231 cells were transfected with miR-15, 16, 17 and 221 followed by compound treatment. After 24 h of incubation, cells were processed for cell cycle analysis and histograms obtained by FCS Express software.
(TIF)

S3 Fig Effect of C-10 on apoptosis. The expression of proapoptotic and antiapoptotic proteins in compound treated MCF-7 and MDA-MB-231 cells was observed by western blotting.
(TIF)

S4 Fig microRNA target prediction by computational analysis. Softwares like TargetScan, PITA and RNAhybrid were used to predict the binding positionS of each microRNAs-15, 16, 17 and 221 on 3’UTR of its target genes STAT3, Bcl-2 and VEGF.
(DOCX)

## Abbreviations

STAT: signal transducer and activator of transcription
FACS: fluorescence-activated cell sorting
VEGF: vascular endothelial growth factor
UTR: untranslated region
HUVEC: human umbilical vein endothelial cell
