# Supplementary material for: Novel Etoposide Analogue Modulates Expression of Angiogenesis Associated microRNAs and Regulates Cell Proliferation by Targeting STAT3 in Breast Cancer
Source: PLoS One. 2015 Nov 9;10(11):e0142006. doi: 10.1371/journal.pone.0142006 (PMC4638343; doi:10.1371/journal.pone.0142006)
Supplement: S1 Fig — (DOCX) [file pone.0142006.s001.docx]

**S1 figure A: Etoposide**


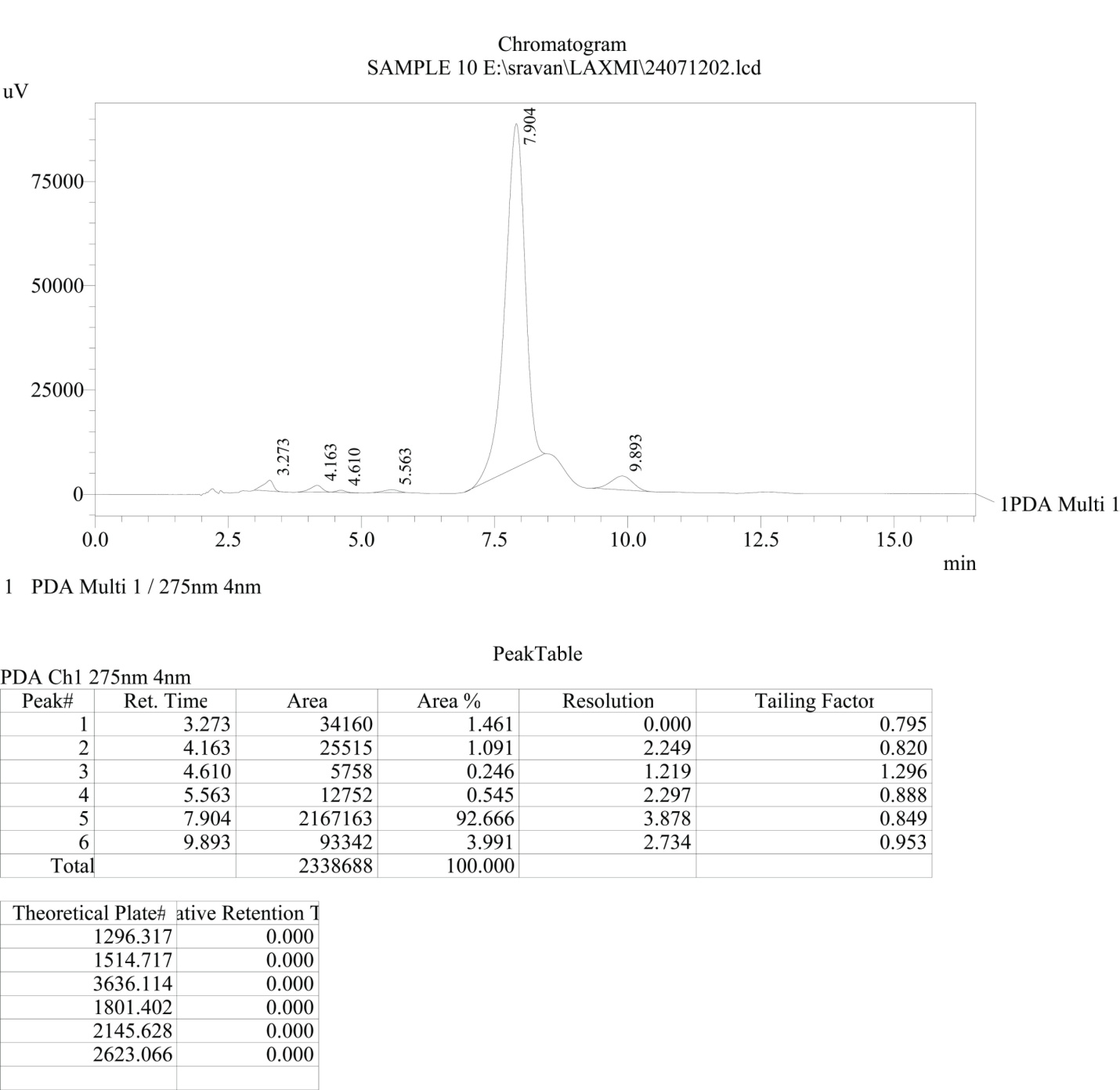


**S1 figure B : C-10**


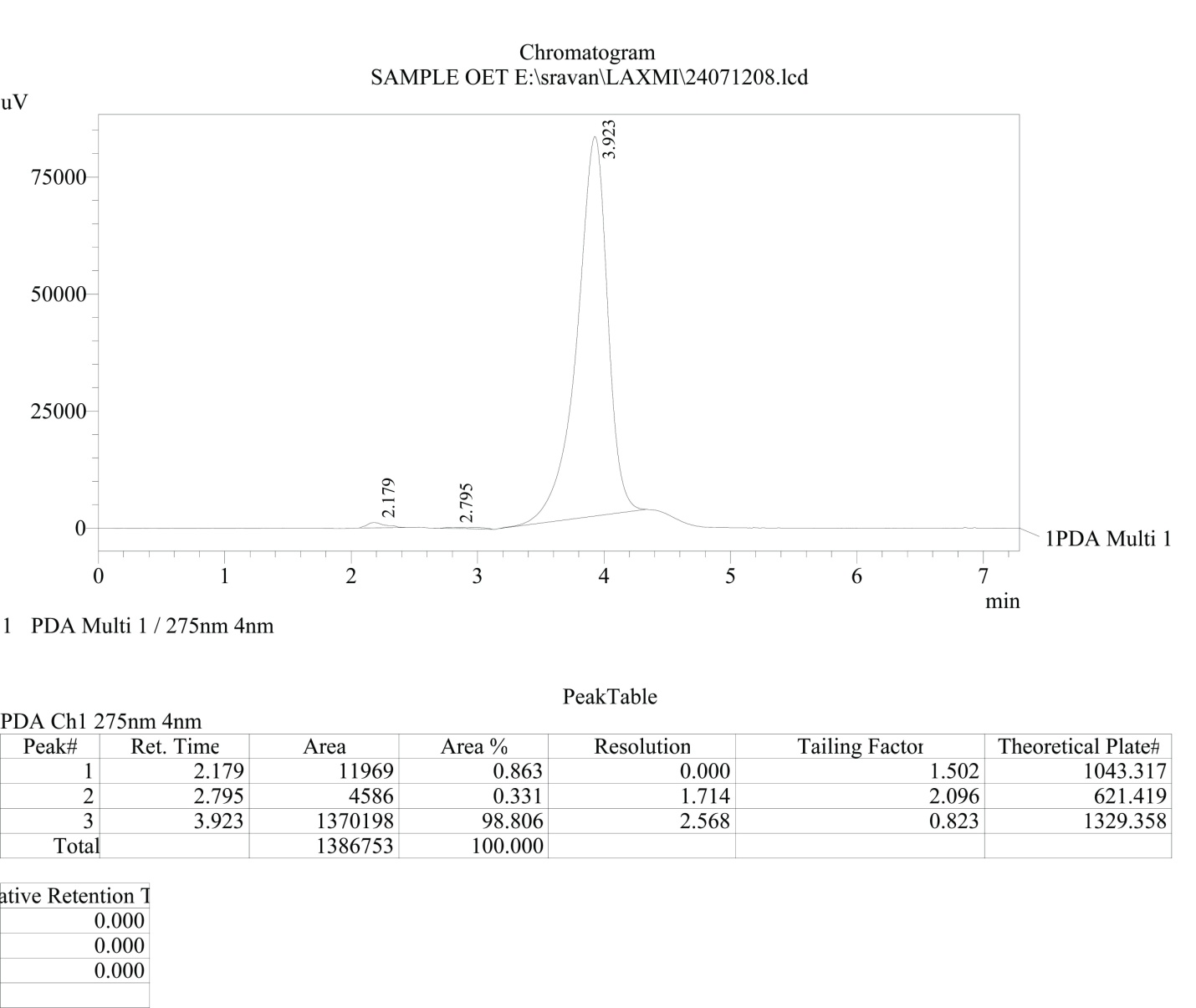


**Supplementary figure 1A&B. HPLC pattern of Etoposide and C-10 compounds.**
