# Supplementary figures and images for: Novel Etoposide Analogue Modulates Expression of Angiogenesis Associated microRNAs and Regulates Cell Proliferation by Targeting STAT3 in Breast Cancer
Source: PLoS One. 2015 Nov 9;10(11):e0142006. doi: 10.1371/journal.pone.0142006 (PMC4638343; doi:10.1371/journal.pone.0142006)

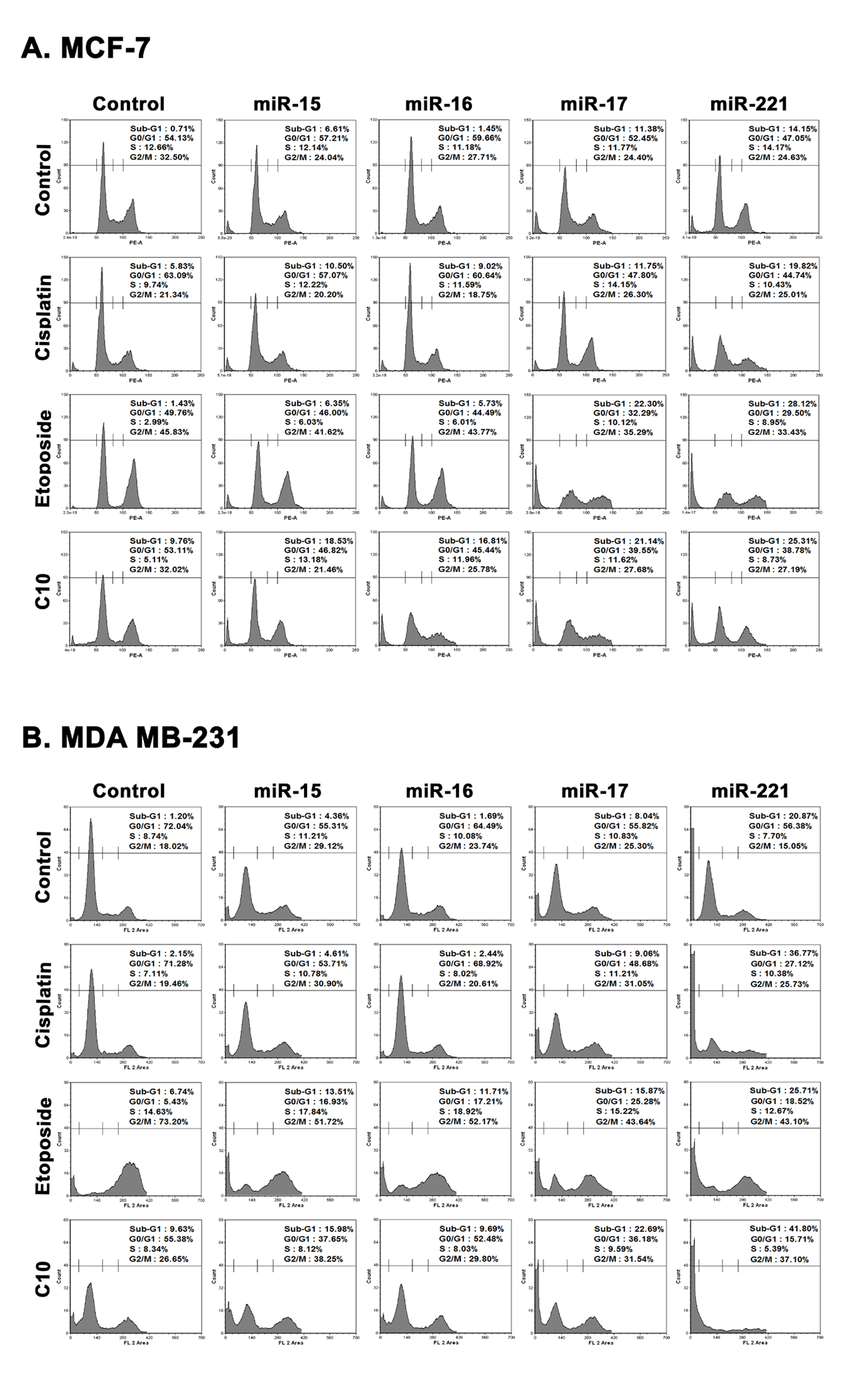

Supplement: S2 Fig — MCF-7 and MDA-MB-231 cells were transfected with miR-15, 16, 17 and 221 followed by compound treatment. After 24 h of incubation, cells were processed for cell cycle analysis and histograms obtained by FCS Express software. (TIF) [file pone.0142006.s002.tif]

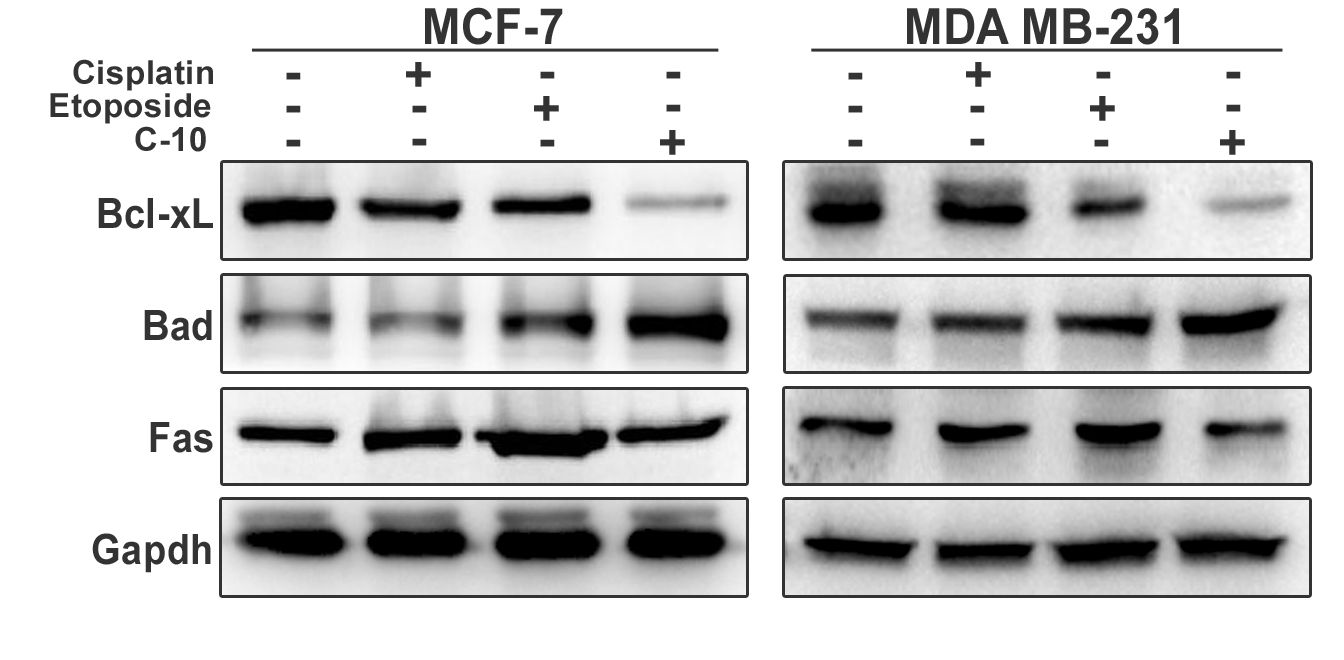

Supplement: S3 Fig — The expression of proapoptotic and antiapoptotic proteins in compound treated MCF-7 and MDA-MB-231 cells was observed by western blotting. (TIF) [file pone.0142006.s003.tif]
