# Supplementary material for: Novel Etoposide Analogue Modulates Expression of Angiogenesis Associated microRNAs and Regulates Cell Proliferation by Targeting STAT3 in Breast Cancer
Source: PLoS One. 2015 Nov 9;10(11):e0142006. doi: 10.1371/journal.pone.0142006 (PMC4638343; doi:10.1371/journal.pone.0142006)

| Position 2529-2536 of BCL2 3' UTR [hsa-miR-15a-5p](http://www.mirbase.org/cgi-bin/mirna_entry.pl?acc=hsa-miR-15a-5p) | 5' ...GAAUAUCCAAUCCUGUGCUGCUA...             \|\|\|      \|\|\|\|\|\|\|  3'    GUGUUUGGUAAUAC-ACGACGAU | 8mer | -0.27 | 94 | -0.25 | 6.636 | 0.90 |
| --- | --- | --- | --- | --- | --- | --- | --- |
| Position 2529-2536 of BCL2 3' UTR [hsa-miR-16-5p](http://www.mirbase.org/cgi-bin/mirna_entry.pl?acc=hsa-miR-16-5p) | 5'    ...GAAUAUCCAAUCCUGUGCUGCUA...           \|\|\|\|\|         \|\|\|\|\|\|\|  3'    GCGGUUAUAAAUGC----ACGACGAU | 8mer | -0.27 | 93 | -0.25 | 6.636 | 0.90 |
| Position 3113-3119 of BCL2 3' UTR [hsa-miR-17-5p](http://www.mirbase.org/cgi-bin/mirna_entry.pl?acc=hsa-miR-17-5p) | 5'   ...UUAAAUAGAGUAUAUGCACUUUC...                        \|\|\|\|\|\|\|  3'      GAUGGACGUGACAUUCGUGAAAC | 7mer-m8 | -0.02 | 58 | -0.02 | 1.419 | < 0.1 |
| Position 3466-3472 of BCL2 3' UTR [hsa-miR-221-3p](http://www.mirbase.org/cgi-bin/mirna_entry.pl?acc=hsa-miR-221-3p) | 5' ...GCUUAUCAUCUAAAGAUGUAGCU...                      \|\|\|\|\|\|\|  3'    CUUUGGGUCGUCUGUUACAUCGA | 7mer-m8 | -0.06 | 51 | -0.05 | 0.166 | < 0.1 |

| Position 276-283 of VEGFA 3' UTR [hsa-miR-15a-5p](http://www.mirbase.org/cgi-bin/mirna_entry.pl?acc=hsa-miR-15a-5p) | 5'  ...CCAUUUUAUUUUUCUUGCUGCUA...                       \|\|\|\|\|\|\|  3'      GUGUUUGGUAAUACACGACGAU | 8mer | -0.52 | 99 | -0.52 | 3.981 | 0.82 |
| --- | --- | --- | --- | --- | --- | --- | --- |
| Position 276-283 of VEGFA 3' UTR [hsa-miR-16-5p](http://www.mirbase.org/cgi-bin/mirna_entry.pl?acc=hsa-miR-16-5p) | 5' ...CCAUUUUAUUUUUCUUGCUGCUA...             \|\|\|\|\|    \|\|\|\|\|\|\|  3'    GCGGUUAUAAAUGC-ACGACGAU | 8mer | -0.55 | 99 | -0.55 | 3.981 | 0.82 |
| Position 177-183 of VEGFA 3' UTR [hsa-miR-17-5p](http://www.mirbase.org/cgi-bin/mirna_entry.pl?acc=hsa-miR-17-5p) | 5'   ...GAGACUCUGCGCAGAGCACUUUG...                        \|\|\|\|\|\|\|  3'      GAUGGACGUGACAUUCGUGAAAC | 7mer-m8 | -0.13 | 91 | -0.13 | 4.122 | 0.73 |

**TargetScan Software**

| Position 542-548 of STAT3 3' UTR [hsa-miR-17-5p](http://www.mirbase.org/cgi-bin/mirna_entry.pl?acc=hsa-miR-17-5p) | 5'   ...CAUACUCCUGGCAUUGCACUUUU...                        \|\|\|\|\|\|\|  3'      GAUGGACGUGACAUUCGUGAAAC | 7mer-m8 | -0.22 | 95 | -0.20 | 4.423 | 0.73 |
| --- | --- | --- | --- | --- | --- | --- | --- |
| Position 2199-2205 of STAT3 3' UTR [hsa-miR-221-3p](http://www.mirbase.org/cgi-bin/mirna_entry.pl?acc=hsa-miR-221-3p) | 5'   ...GGUCUUAACUCUGAUUGUAGCAA...                        \|\|\|\|\|\|   3'     CUUUGGGUCGUCUGUUACAUCGA | 7mer-1A | -0.11 | 71 | -0.10 | 1.815 | 0.11 |

**PITA Software (Segal Lab)**

| **Organism** | **RefSeq** | **Gene Name** | **microRNA** | **Sites** | **Score** |
| --- | --- | --- | --- | --- | --- |
| **Human** | **NM_003150;NM_139276;NM_213662** | **STAT3** | **hsa-miR-15a** | **1** | **-4.09** |
| **Human** | **NM_003150;NM_139276;NM_213662** | **STAT3** | **hsa-miR-16** | **1** | **-5.25** |
| **Human** | **NM_003150;NM_139276;NM_213662** | **STAT3** | **hsa-miR-17** | **3** | **-9.82** |
| **Human** | **NM_003150;NM_139276;NM_213662** | **STAT3** | **hsa-miR-221** | **1** | **-7.67** |

| **Organism** | **RefSeq** | **Gene Name** | **microRNA** | **Sites** | **Score** |
| --- | --- | --- | --- | --- | --- |
| **Human** | **NM_001025366;NM_001025367;NM_001025368;NM_001025369;NM_001025370;NM_001033756;NM_003376** | **VEGF** | **hsa-miR-15a** | **1** | **-9.33** |
| **Human** | **NM_001025366;NM_001025367;NM_001025368;NM_001025369;NM_001025370;NM_001033756;NM_003376** | **VEGF** | **hsa-miR-16** | **1** | **-15.51** |
| **Human** | **NM_001025366;NM_001025367;NM_001025368;NM_001025369;NM_001025370;NM_001033756;NM_003376** | **VEGF** | **hsa-miR-17** | **1** | **-10.73** |

| **Organism** | **RefSeq** | **Gene Name** | **microRNA** | **Sites** | **Score** |
| --- | --- | --- | --- | --- | --- |
| **Human** | **NM_000633** | **BCL2** | **hsa-miR-15a** | **4** | **-9.65** |
| **Human** | **NM_000633** | **BCL2** | **hsa-miR-16** | **4** | **-12.51** |
| **Human** | **NM_000633** | **BCL2** | **hsa-miR-17** | **3** | **-4.90** |
| **Human** | **NM_000633** | **BCL2** | **hsa-miR-221** | **2** | **-4.22** |

**RNAhybrid Software**

**STAT3:**

**miR-15**

Version: RNAhybrid 2.2

Command line:/vol/bioapps/bin/RNAhybrid.bin -n 22 -q /var/bibiserv2/anonymous/rnahybrid/25/15/17/bibiserv2_2015-08-25_151728_sWTFE/rnahybrid_input_mirna_sequences.file -g all -b 1 -m 4978 -s 3utr_human -t /var/bibiserv2/anonymous/rnahybrid/25/15/17/bibiserv2_2015-08-25_151728_sWTFE/rnahybrid_input_target_rna_sequences_.file

searching

dataset: 1

mde of hsa-miR-15a-5p: -42.200001

Individual hits

------------------------------------------------------------

dataset: 1

target: gi|47080104|ref|NM_139276.2|

length: 4978

miRNA : hsa-miR-15a-5p

length: 22

mfe: -22.5 kcal/mol

p-value: 1.000000e+00

position 1506

target 5' C AU A U 3'

CGAGCCA UGUG UGCU

GUUUGGU ACAC ACGA

miRNA 3' GU AAU G U 5'

------------------------------------------------------------


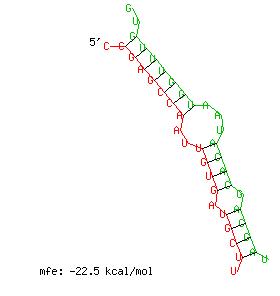


**miR-16**

Version: RNAhybrid 2.2

Command line:/vol/bioapps/bin/RNAhybrid.bin -n 22 -q /var/bibiserv2/anonymous/rnahybrid/25/15/22/bibiserv2_2015-08-25_152218_8KpRf/rnahybrid_input_mirna_sequences.file -g all -b 1 -m 4978 -s 3utr_human -t /var/bibiserv2/anonymous/rnahybrid/25/15/22/bibiserv2_2015-08-25_152218_8KpRf/rnahybrid_input_target_rna_sequences_.file

searching

dataset: 1

mde of hsa-miR-16-5p: -43.799999

Individual hits

------------------------------------------------------------

dataset: 1

target: gi|47080104|ref|NM_139276.2|

length: 4978

miRNA : hsa-miR-16-5p

length: 22

mfe: -23.5 kcal/mol

p-value: 1.000000e+00

position 1145

target 5' G CUGGAGGAGAGAAU GA U 3'

GCCGAUG CGUG GCUG

CGGUUAU GCAC CGAU

miRNA 3' G AAAU GA 5'

------------------------------------------------------------


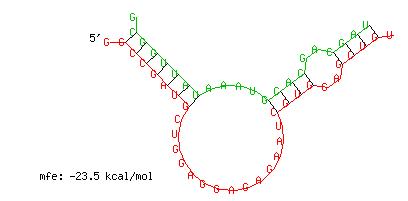


**miR-17**

Version: RNAhybrid 2.2

Command line:/vol/bioapps/bin/RNAhybrid.bin -n 23 -q /var/bibiserv2/anonymous/rnahybrid/25/15/29/bibiserv2_2015-08-25_152928_FjAxp/rnahybrid_input_mirna_sequences.file -g all -b 1 -m 4978 -s 3utr_human -t /var/bibiserv2/anonymous/rnahybrid/25/15/29/bibiserv2_2015-08-25_152928_FjAxp/rnahybrid_input_target_rna_sequences_.file

searching

dataset: 1

mde of hsa-miR-17-5p: -46.700001

Individual hits

------------------------------------------------------------

dataset: 1

target: gi|47080104|ref|NM_139276.2|

length: 4978

miRNA : hsa-miR-17-5p

length: 23

mfe: -30.9 kcal/mol

p-value: 1.000000e+00

position 2942

target 5' U UU A 3'

CUGCCUGU CUGUAAGCA

GAUGGACG GACAUUCGU

miRNA 3' U GAAAC 5'

------------------------------------------------------------


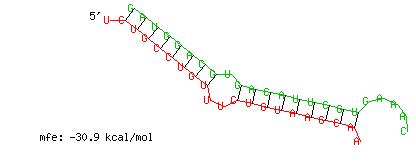


**miR-221**

Version: RNAhybrid 2.2

Command line:/vol/bioapps/bin/RNAhybrid.bin -n 22 -q /var/bibiserv2/anonymous/rnahybrid/25/15/32/bibiserv2_2015-08-25_153227_lhnq8/rnahybrid_input_mirna_sequences.file -g all -b 1 -m 4978 -s 3utr_human -t /var/bibiserv2/anonymous/rnahybrid/25/15/32/bibiserv2_2015-08-25_153227_lhnq8/rnahybrid_input_target_rna_sequences_.file

searching

dataset: 1

mde of hsa-miR-221-5p: -42.100002

Individual hits

------------------------------------------------------------

dataset: 1

target: gi|47080104|ref|NM_139276.2|

length: 4978

miRNA : hsa-miR-221-5p

length: 22

mfe: -23.6 kcal/mol

p-value: 1.000000e+00

position 3725

target 5' U UUCAGAG U 3'

GAUCUGC UGU GCUAGGU

UUAGAUG ACA CGGUCCA

miRNA 3' U UA UA 5'

------------------------------------------------------------


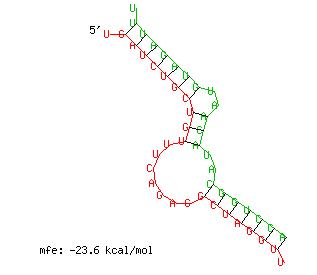


**Bcl-2:**

**miR-15**

Version: RNAhybrid 2.2

Command line:/vol/bioapps/bin/RNAhybrid.bin -n 22 -q /var/bibiserv2/anonymous/rnahybrid/25/15/40/bibiserv2_2015-08-25_154059_vz0yu/rnahybrid_input_mirna_sequences.file -g all -b 1 -m 6492 -s 3utr_human -t /var/bibiserv2/anonymous/rnahybrid/25/15/40/bibiserv2_2015-08-25_154059_vz0yu/rnahybrid_input_target_rna_sequences_.file

searching

dataset: 1

mde of hsa-miR-15a-5p: -42.200001

Individual hits

------------------------------------------------------------

dataset: 1

target: gi|72198188|ref|NM_000633.2|

length: 6492

miRNA : hsa-miR-15a-5p

length: 22

mfe: -24.3 kcal/mol

p-value: 1.000000e+00

position 3728

target 5' A U AUCC U 3'

AUA CCA UGUGCUGCUA

UGU GGU ACACGACGAU

miRNA 3' G UU AAU 5'

------------------------------------------------------------


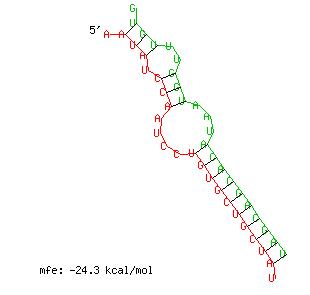


**miR-16**

Version: RNAhybrid 2.2

Command line:/vol/bioapps/bin/RNAhybrid.bin -n 22 -q /var/bibiserv2/anonymous/rnahybrid/25/15/43/bibiserv2_2015-08-25_154300_5WMF5/rnahybrid_input_mirna_sequences.file -g all -b 1 -m 6492 -s 3utr_human -t /var/bibiserv2/anonymous/rnahybrid/25/15/43/bibiserv2_2015-08-25_154300_5WMF5/rnahybrid_input_target_rna_sequences_.file

searching

dataset: 1

mde of hsa-miR-16-5p: -43.799999

Individual hits

------------------------------------------------------------

dataset: 1

target: gi|72198188|ref|NM_000633.2|

length: 6492

miRNA : hsa-miR-16-5p

length: 22

mfe: -24.1 kcal/mol

p-value: 1.000000e+00

position 3732

target 5' U CC U 3'

CCAAU UGUGCUGCUA

GGUUA GCACGACGAU

miRNA 3' GC UAAAU 5'

------------------------------------------------------------


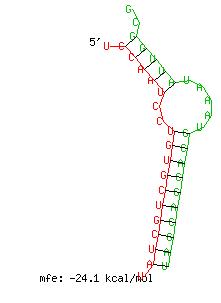


**miR-17**

Version: RNAhybrid 2.2

Command line:/vol/bioapps/bin/RNAhybrid.bin -n 23 -q /var/bibiserv2/anonymous/rnahybrid/25/15/44/bibiserv2_2015-08-25_154454_ZAgyH/rnahybrid_input_mirna_sequences.file -g all -b 1 -m 6492 -s 3utr_human -t /var/bibiserv2/anonymous/rnahybrid/25/15/44/bibiserv2_2015-08-25_154454_ZAgyH/rnahybrid_input_target_rna_sequences_.file

searching

dataset: 1

mde of hsa-miR-17-5p: -46.700001

Individual hits

------------------------------------------------------------

dataset: 1

target: gi|72198188|ref|NM_000633.2|

length: 6492

miRNA : hsa-miR-17-5p

length: 23

mfe: -25.1 kcal/mol

p-value: 1.000000e+00

position 3951

target 5' G G CUUUU GU G 3'

CUG CUGC GCUGUGGG UUU

GAU GACG UGACAUUC GAA

miRNA 3' G GU AC 5'

------------------------------------------------------------


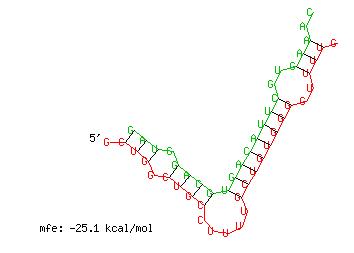


**miR-221**

Version: RNAhybrid 2.2

Command line:/vol/bioapps/bin/RNAhybrid.bin -n 22 -q /var/bibiserv2/anonymous/rnahybrid/25/15/46/bibiserv2_2015-08-25_154627_AWwW1/rnahybrid_input_mirna_sequences.file -g all -b 1 -m 6492 -s 3utr_human -t /var/bibiserv2/anonymous/rnahybrid/25/15/46/bibiserv2_2015-08-25_154627_AWwW1/rnahybrid_input_target_rna_sequences_.file

searching

dataset: 1

mde of hsa-miR-221-5p: -42.100002

Individual hits

------------------------------------------------------------

dataset: 1

target: gi|72198188|ref|NM_000633.2|

length: 6492

miRNA : hsa-miR-221-5p

length: 22

mfe: -23.6 kcal/mol

p-value: 1.000000e+00

position 5115

target 5' U CAA UAUGA U 3'

UCUACAUUGU GCU AGGU

AGAUGUAACA CGG UCCA

miRNA 3' UUU UA 5'

------------------------------------------------------------


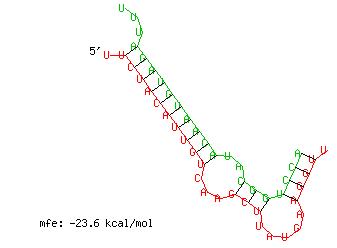


**VEGFA:**

**miR-15**

Version: RNAhybrid 2.2

Command line:/vol/bioapps/bin/RNAhybrid.bin -n 22 -q /var/bibiserv2/anonymous/rnahybrid/25/15/51/bibiserv2_2015-08-25_155155_RgE0g/rnahybrid_input_mirna_sequences.file -g all -b 1 -m 3677 -s 3utr_human -t /var/bibiserv2/anonymous/rnahybrid/25/15/51/bibiserv2_2015-08-25_155155_RgE0g/rnahybrid_input_target_rna_sequences_.file

searching

dataset: 1

mde of hsa-miR-15a-5p: -42.200001

Individual hits

------------------------------------------------------------

dataset: 1

target: gi|284172447|ref|NM_001025366.2|

length: 3677

miRNA : hsa-miR-15a-5p

length: 22

mfe: -25.7 kcal/mol

p-value: 1.000000e+00

position 1256

target 5' U CC CCCUGA G 3'

CAAGCCAU UGUGUGC UGC

GUUUGGUA AUACACG ACG

miRNA 3' GU AU 5'

----------------------------------------------------------


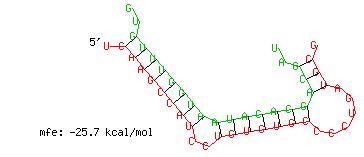


**miR-16**

Version: RNAhybrid 2.2

Command line:/vol/bioapps/bin/RNAhybrid.bin -n 22 -q /var/bibiserv2/anonymous/rnahybrid/25/15/55/bibiserv2_2015-08-25_155558_mTU81/rnahybrid_input_mirna_sequences.file -g all -b 1 -m 3677 -s 3utr_human -t /var/bibiserv2/anonymous/rnahybrid/25/15/55/bibiserv2_2015-08-25_155558_mTU81/rnahybrid_input_target_rna_sequences_.file

searching

dataset: 1

mde of hsa-miR-16-5p: -43.799999

Individual hits

------------------------------------------------------------

dataset: 1

target: gi|284172447|ref|NM_001025366.2|

length: 3677

miRNA : hsa-miR-16-5p

length: 22

mfe: -27.4 kcal/mol

p-value: 1.000000e+00

position 1995

target 5' U UUU UU U A 3'

CGCCA UAUUU C UGCUGCUA

GCGGU AUAAA G ACGACGAU

miRNA 3' U U C 5'

-----------------------------------------------------------


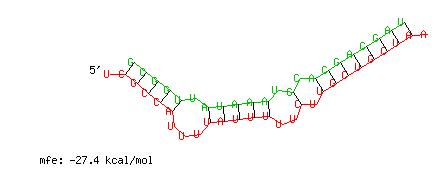


**miR-17**

Version: RNAhybrid 2.2

Command line:/vol/bioapps/bin/RNAhybrid.bin -n 23 -q /var/bibiserv2/anonymous/rnahybrid/25/15/57/bibiserv2_2015-08-25_155738_hzGzV/rnahybrid_input_mirna_sequences.file -g all -b 1 -m 3677 -s 3utr_human -t /var/bibiserv2/anonymous/rnahybrid/25/15/57/bibiserv2_2015-08-25_155738_hzGzV/rnahybrid_input_target_rna_sequences_.file

searching

dataset: 1

mde of hsa-miR-17-5p: -46.700001

Individual hits

------------------------------------------------------------

dataset: 1

target: gi|284172447|ref|NM_001025366.2|

length: 3677

miRNA : hsa-miR-17-5p

length: 23

mfe: -29.7 kcal/mol

p-value: 1.000000e+00

position 1901

target 5' G U A G 3'

AC CUGCGC GAGCACUUUG

UG GACGUG UUCGUGAAAC

miRNA 3' GA ACA 5'

------------------------------------------------------------


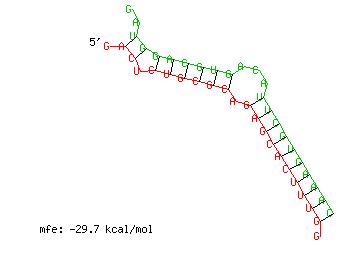


**miR-221**

Version: RNAhybrid 2.2

Command line:/vol/bioapps/bin/RNAhybrid.bin -n 22 -q /var/bibiserv2/anonymous/rnahybrid/25/15/59/bibiserv2_2015-08-25_155910_1h2bK/rnahybrid_input_mirna_sequences.file -g all -b 1 -m 3677 -s 3utr_human -t /var/bibiserv2/anonymous/rnahybrid/25/15/59/bibiserv2_2015-08-25_155910_1h2bK/rnahybrid_input_target_rna_sequences_.file

searching

dataset: 1

mde of hsa-miR-221-5p: -42.100002

Individual hits

------------------------------------------------------------

dataset: 1

target: gi|284172447|ref|NM_001025366.2|

length: 3677

miRNA : hsa-miR-221-5p

length: 22

mfe: -22.6 kcal/mol

p-value: 1.000000e+00

position 2351

target 5' G G AG G 3'

GAAU UGCA GCCAGG

UUUA AUGU CGGUCC

miRNA 3' G AACAUA A 5'

------------------------------------------------------------


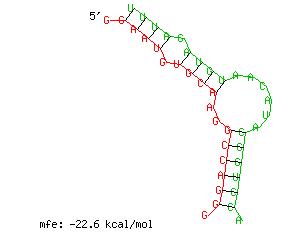

Supplement: S4 Fig — Softwares like TargetScan, PITA and RNAhybrid were used to predict the binding positionS of each microRNAs-15, 16, 17 and 221 on 3’UTR of its target genes STAT3, Bcl-2 and VEGF. (DOCX) [file pone.0142006.s004.docx]
